# Dual CHK1 and WNT Inhibition as a Synergistic Therapy for Gastrointestinal Neuroendocrine Carcinoma

**DOI:** 10.1158/2767-9764.CRC-26-0351

**Published:** 2026-10-19

**Authors:** Hirokazu Kimura, Yoshihiko Sato, Shinichi Yachida

**Affiliations:** 1 Department of Cancer Genome Informatics, Graduate School of Medicine, The University of Osaka, Osaka, Japan.; 2 Integrated Frontier Research for Medical Science Division, Institute for Open and Transdisciplinary Research Initiatives (OTRI), The University of Osaka, Osaka, Japan.; 3 Japan Agency for Medical Research and Development -Core Research for Evolutional Science and Technology (AMED-CREST), Tokyo, Japan.

## Abstract

Gastrointestinal neuroendocrine carcinoma (GIS-NEC) is a highly aggressive malignancy with limited therapeutic options. To identify a therapeutic target, we performed transcriptomic profiling of 34 GIS-NEC and 30 well-differentiated gastrointestinal neuroendocrine tumors (GIS-NET) and found that checkpoint kinase 1 (*CHEK1*) is significantly overexpressed in GIS-NEC. Although *CHEK1* expression was not prognostic across overall GIS-NEC, it was associated with poor outcome specifically in pancreatic NEC, in which it correlated with replication stress–associated genes, including cell division cycle 6 (*CDC6*) and claspin (*CLSPN*), both at the intercase level and across spatially distinct regions within individual tumors. Pharmacologic inhibition of checkpoint kinase 1 (CHK1) using prexasertib (LY2606368) suppressed cell proliferation, induced cell-cycle arrest, and increased DNA damage in most GIS-NEC cell lines, recapitulating the effects of *CHEK1* knockdown. Antitumor efficacy was further confirmed *in vivo*. However, pancreatic NEC cell lines exhibited relative resistance to CHK1 inhibition, and transcriptomic analysis revealed enrichment of WNT signaling–related genes in these cells. Low-density lipoprotein receptor–related protein 8 (*LRP8*) was highly expressed in pancreatic NEC cases with high *CHEK1* expression. WNT inhibition suppressed *MYC*-dependent transcriptional programs and downregulated replication-associated genes, including *CDC6*, thereby impairing replication capacity. Combined WNT and CHK1 inhibition further exacerbated replication stress and DNA damage, even at low CHK1 inhibitor doses. Collectively, these findings identify CHK1 as a therapeutic vulnerability in GIS-NEC and indicate that WNT–MYC signaling contributes to resistance by sustaining replication programs. Dual targeting of CHK1 and WNT–MYC pathways may therefore represent a promising therapeutic strategy, particularly in pancreatic NEC.

**Significance:**

*CHEK1* is overexpressed in GIS-NEC and represents a therapeutic vulnerability. In tumors in which CHK1 inhibition alone is ineffective, combined CHK1 and WNT inhibition exerts synergistic effects, enhancing replication stress and DNA damage.

## Introduction

Neuroendocrine neoplasms (NEN) arise in various organs, with the gastrointestinal system representing one of the most common sites. According to the 2019 World Health Organization classification, NENs are categorized into well-differentiated neuroendocrine tumors (NET) and poorly differentiated neuroendocrine carcinomas (NEC) based on differentiation status, mitotic count, and Ki-67 index (1–3). Gastrointestinal NEC (GIS-NEC) is a rare but highly aggressive malignancy with an incidence of approximately five cases per million population (4–6). Platinum-based chemotherapy, similar to that used for small cell lung cancer (SCLC), remains the standard treatment. However, therapeutic resistance frequently develops, and the median overall survival (OS) is approximately 12 months (7). Despite ongoing clinical trials of agents such as immune checkpoint inhibitors, effective treatment options remain limited (8–12).

Recent molecular profiling studies have begun to reveal the genomic landscape of GIS-NEC. Our previous multiomic analysis of 115 gastrointestinal NEN (GIS-NEN) tumors identified organ-specific driver mutations, transcriptional programs, and novel fusion genes, highlighting the biological heterogeneity of these tumors (13). In SCLC, molecular subtypes defined by transcriptional regulators such as *ASCL1*, *POU2F3*, *YAP1*, and *NEUROD1* have been proposed, suggesting subtype-specific therapeutic vulnerabilities (9, 14, 15). Supporting this concept, our previous work identified distinct molecular subtypes of GIS-NEC, including a *PTF1A*-type pancreatic NEC (13). However, therapeutic targets for these molecular subgroups remain largely undefined.

Targeting replication stress has recently emerged as a promising anticancer strategy (16). The ataxia-telangiectasia and Rad3-related (ATR)–checkpoint kinase 1 (CHK1) signaling axis, with CHK1 encoded by the *CHEK1* gene, plays a central role in maintaining replication fork stability and coordinating DNA damage responses during the S- and G_2_–M-phases of the cell cycle (16–18). Inhibition of CHK1 disrupts these processes, exacerbates replication stress, and ultimately induces cell death. Prexasertib (LY2606368), a selective CHK1 inhibitor, has demonstrated antitumor activity in several preclinical models, including SCLC, which, like GIS-NEC, is characterized by frequent *TP53* and *RB1* alterations (17, 19). However, clinical trials of prexasertib in extensive-stage SCLC failed to show meaningful clinical benefit (20), although CHK1 inhibition has shown clinical activity in another *TP53*-mutant malignancy, high-grade serous ovarian carcinoma (HGSOC; refs. 21, 22). These observations indicate that responses to CHK1 inhibition are context dependent and not predicted by *TP53* alterations alone. Although GIS-NEC shares molecular features with SCLC, it also exhibits distinct characteristics, including site-specific mutations in genes such as *APC* and *KRAS* (13, 23). The therapeutic relevance of CHK1 inhibition in GIS-NEC therefore remains unclear, and no preclinical studies have systematically evaluated its efficacy.

In this study, we demonstrate that *CHEK1* is markedly upregulated in GIS-NEC and that its inhibition induces cell-cycle arrest, DNA damage, and apoptosis. We further identify WNT signaling as a pathway that modulates sensitivity to CHK1 inhibition, particularly in pancreatic NEC, in which high *CHEK1* expression is associated with poor prognosis. Combined inhibition of CHK1 and WNT signaling enhanced antitumor effects in resistant models. These findings identify CHK1 as a potential therapeutic target in GIS-NEC and support combined targeting of CHK1 and WNT pathways as a promising strategy, especially in pancreatic NEC.

## Materials and Methods

### Cell culture and reagents

Six GIS-NEC cell lines, one SCLC cell line, one gastrointestinal NET (GIS-NET) cell line, and nine pancreatic ductal adenocarcinoma (PDAC) cell lines were used in this study (Supplementary Table S1). A99 cells were established at a previous institution (24) and subsequently transferred to the current laboratory. Other cell lines were obtained from the indicated cell banks or provided by a collaborator, as detailed in Supplementary Table S1. Cell line authentication was not performed in our laboratory. *Mycoplasma* testing was performed for three cell lines, as indicated in Supplementary Table S1. Cells were used within the indicated ranges of passages after thawing. The biological sex and donor age of each cell line are provided in Supplementary Table S1. Reagents and antibodies used in this study are listed in Supplementary Table S2.

### Immunohistochemistry

Immunohistochemistry (IHC) for CHK1 in human GIS-NEC specimens was performed using a protocol adapted from a previously described method (25) with modifications. Antigen retrieval was performed using Dako Target Retrieval Solution, pH 9.0 (cat. #S2367, Dako), at 121°C for 10 minutes. After deparaffinization and heat-induced antigen retrieval, endogenous peroxidase activity was quenched with Dako REAL Peroxidase-Blocking Solution (cat. #S2023, Dako) for 10 minutes, followed by blocking with G-Block (cat. #GB-01, GenoStaff) for 10 minutes. The sections were then incubated with an anti-Chk1 (2G1D5) antibody (Cell Signaling Technology, #2360; 1:600) for 16 hours at 4°C, followed by incubation with horseradish peroxidase–conjugated goat anti–mouse IgG polymer reagent (ImmPRESS, cat. #30119) for 30 minutes and 3,3′-diaminobenzidine (DAB) development (Histofine, cat. #425011, Nichirei). Previously generated IHC slides for synaptophysin and Ki-67 from our published GIS-NEC cohort (13) were also used for comparison.

For mouse tumor samples, IHC was performed as previously described (26) with minor modifications. Briefly, formalin-fixed, paraffin-embedded tumors were sectioned at 4 μm, followed by antigen retrieval in 0.01 mol/L citrate buffer (pH 6.0) at 121°C for 10 minutes. Sections were treated with 3% H_2_O_2_ for 15 minutes, blocked with Blocking One Histo (Nacalai Tesque, cat. #06349-64) for 10 minutes, and incubated with primary antibodies overnight at 4°C, followed by secondary antibody incubation and DAB detection. Antibody details are provided in Supplementary Table S2. Images were acquired using a BZ-X800 microscope (Keyence), and the Ki-67 labeling index was calculated as the percentage of positive cells among >5,000 cells across 10 tumor areas per group.

### *TP53* alteration status

*TP53* alteration data for the six GIS-NEC cell lines were obtained from published studies (13, 24, 27), the International Agency for Research on Cancer *TP53* Database (28), and Cellosaurus (29). The clinical significance of *TP53* alterations was assessed using the Franklin by Genoox clinical variant interpretation platform (https://franklin.genoox.com/clinical-db/home), and all identified alterations were classified as tier I somatic variants. ClinVar pathogenicity classifications are also provided in Supplementary Table S3 as supplementary annotation.

### RNA sequencing data analysis

Publicly available, deidentified RNA sequencing (RNA-seq) data were obtained from the the Japanese Genotype-phenotype Archive (JGA; accession: JGAS000359; ref. 13). The dataset included 34 GIS-NEC and 30 GIS-NET samples (male, *n* = 37; female, *n* = 27; median age, 60 years; range, 23–86 years), as listed in Supplementary Table S4. Read count estimation and transcripts per million (TPM) normalization were performed as previously described (13). RNA-seq data for six GIS-NEC cell lines are available from the Gene Expression Omnibus repository under accession number GSE190618 (30).

### Quantitative RT-PCR

Total RNA was extracted using the AllPrep DNA/RNA Mini Kit (QIAGEN), and cDNA was synthesized with the SuperScript VILO cDNA Synthesis Kit (Invitrogen), according to the respective manufacturer’s protocol. Quantitative RT-PCR was performed on a LightCycler 96 system (Roche) using the iTaq Universal SYBR Green Supermix (Bio-Rad). Gene expression was normalized to *GAPDH* or *ACTB*. Primer sequences are listed in Supplementary Table S5.

### Xenograft tumor formation assay

Six-week-old female BALB/c nude mice (RRID: IMSR_CRL:194, Charles River Laboratories) were subcutaneously injected with 5 × 10^6^ TYUC-1 cells suspended in 50 μL of medium mixed with 50 μL of Matrigel (cat. #354234, BD Biosciences). When tumors reached 100 to 150 mm^3^, mice were randomized into vehicle control and prexasertib groups. Prexasertib (10 mg/kg) was injected intraperitoneally twice daily on days 1 and 2 each week for 4 weeks (40 mg/kg/week; refs. 19, 31). Tumor volume was measured three times per week using the formula: (length × width^2^)/2 (19). Mice with dyspnea or >20% weight loss were removed from the study and euthanized. All procedures were approved by the Animal Research Committee of The University of Osaka (number 30-084-003) and conducted in accordance with institutional guidelines. The protocol was prepared in advance but not preregistered. Sample sizes were empirically determined considering the 3R (replacement, reduction, and refinement) principles.

### Kyoto Encyclopedia of Genes and Genomes pathway enrichment analysis

Kyoto Encyclopedia of Genes and Genomes (KEGG; RRID: SCR_012773) pathway enrichment analysis was performed using The R Project for Statistical Computing (version 4.4.2; RRID: SCR_001905) and the Bioconductor (RRID: SCR_006442) package clusterProfiler (RRID: SCR_016884; refs. 32, 33). Gene symbols were converted to Entrez Gene (RRID: SCR_002473) IDs using the bitr function in the org.Hs.eg.db annotation package. Enrichment analysis was conducted using the enrichKEGG function with the organism set to “hsa.”

### Analysis of intratumoral regional variability in gene expression

A phylogenetic tree based on somatic mutation and loss of heterozygosity (LOH) patterns for case NE004 was previously constructed using Randomized Axelerated Maximum Likelihood (RAxML; RRID: SCR_006086; ref. 34) and reported by our group (13). The published tree was used to classify regions without additional phylogenetic reconstruction. Based on their positions in the phylogenetic tree, the 24 sampled regions were classified into 10 phylogenetic groups (A–J), with some groups containing a single sample.

Gene expression variability was assessed across phylogenetically defined groups. Total variance and within-group variance were calculated, and the mean within-group variance was compared with the total variance. Differences across groups were evaluated using one-way analysis of variance (ANOVA). The effect size (η^2^) was calculated as the proportion of total variance explained by between-group differences. The coefficient of variation (CV) was defined as the standard deviation divided by the mean. In addition, a linear mixed-effects model with group included as a random effect was used to estimate the intraclass correlation coefficient (ICC), representing the proportion of variance attributable to between-group differences. These statistical analyses were performed using R (version 4.4.2) with the packages dplyr, ggplot2 (RRID: SCR_014601), and lme4.

### Statistical analysis

Statistical comparisons were performed using JMP version 11 (SAS Institute). Two-group comparisons were performed using the Mann–Whitney *U* test and multiple-group comparisons using the Kruskal–Wallis test. Data from ≥2 independent experiments are presented as mean ± SD. OS was estimated with the Kaplan–Meier analysis and assessed using the generalized Wilcoxon test. Pearson correlation coefficients (*r*) and *P* values were calculated to assess the correlation between gene expression levels. Statistical significance was defined as *P* < 0.05.

## Results

### Genomic analysis of GIS-NEN identifies *CHEK1* as a potential therapeutic target in GIS-NEC

**Figure 1.**
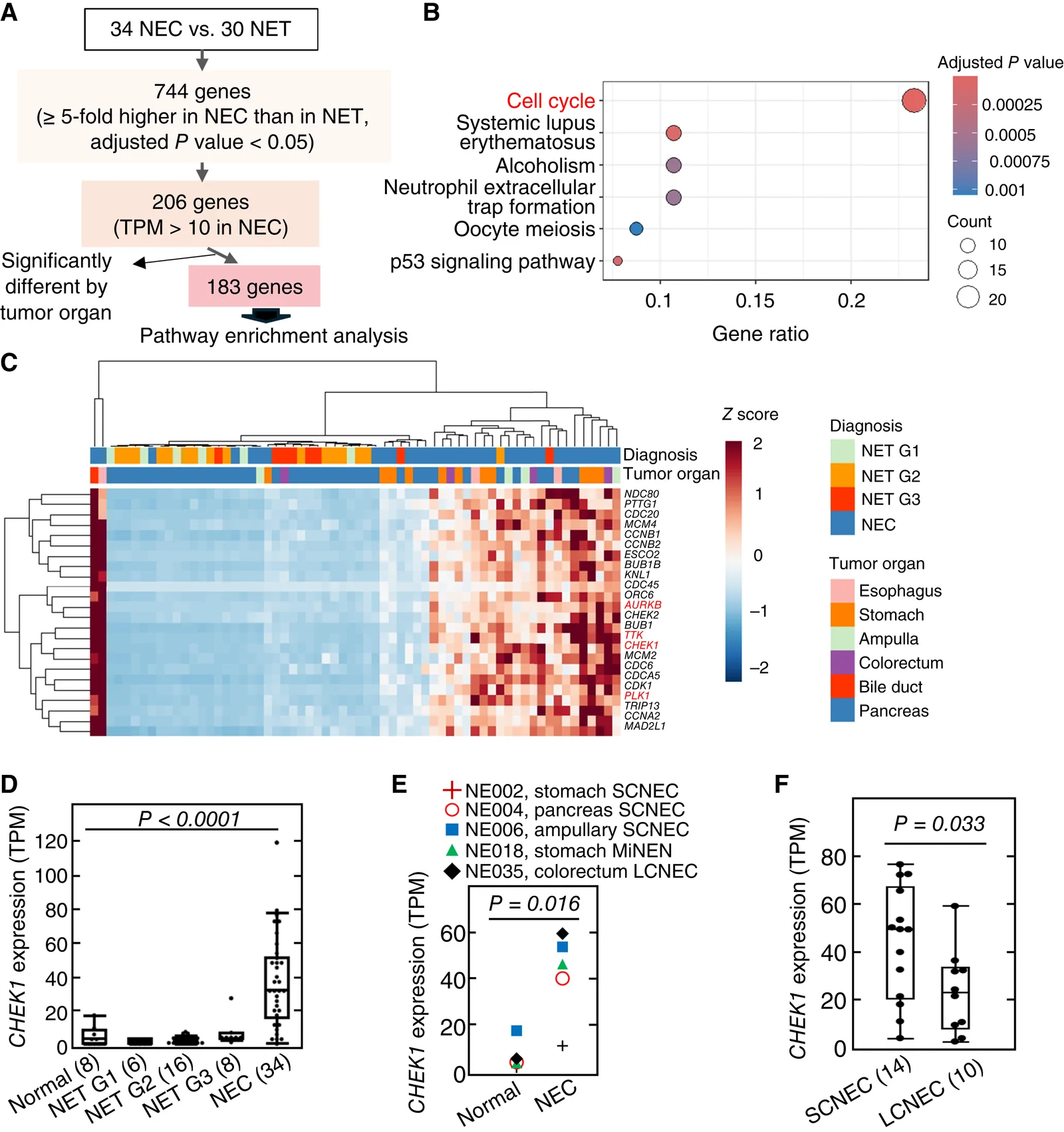
*CHEK1* is highly expressed in GIS-NEC compared with GIS-NET. **A,** Flowchart illustrating the gene selection process for pathway enrichment analysis. A total of 744 genes with ≥5-fold higher expression in GIS-NEC than in GIS-NET (adjusted *P* < 0.05) were extracted. Among these, 206 genes had TPM >10 in GIS-NEC. After excluding genes with significant expression differences by tumor location, a final set of 188 genes was used for KEGG pathway analysis. **B,** Pathway enrichment analysis of the 188 genes shown in A using the KEGG database. **C,** Heatmap showing the expression profiles of 24 genes involved in the cell cycle, identified as the top enriched KEGG pathway in B. **D,** Expression levels of *CHEK1* mRNA in normal tissues from 8 cases and tumor tissues from 64 cases with GIS-NEN were measured by RNA-seq. **E,** Expression levels of *CHEK1* mRNA in paired normal and tumor tissues from the same organ were analyzed in five NEC cases. Data are presented as individual values for each case, highlighting differences in expression between normal and tumor tissues. **F,** Expression levels of *CHEK1* mRNA by histopathologic tumor classification in NEC cases were measured by RNA-seq. Results are presented as box and whisker plots, in which the median is indicated by a line, the box represents the 25th to 75th percentiles, and the whiskers show the 5th to 95th percentiles (**D–F**). *P* values were calculated using the Kruskal–Wallis (**D**) and Mann–Whitney U tests (**E** and **F**). G, grade; MiNEN, mixed neuroendocrine–non-neuroendocrine neoplasm; NET, neuroendocrine tumor.

To identify novel therapeutic targets for GIS-NEC, we analyzed transcriptomic profiles from a previous study of tumor tissues from 30 GIS-NET and 34 GIS-NEC cases (Supplementary Table S4; ref. 13). Clustering analysis identified 183 genes significantly upregulated in GIS-NEC compared with GIS-NET, with a shared expression pattern across tumors arising from different primary organs (Fig. 1A). KEGG pathway enrichment analysis revealed the cell cycle as the most significantly enriched pathway, including 24 of the 183 genes (Fig. 1B). Among these, four genes—aurora kinase B (*AURKB*), *CHEK1*, polo-like kinase 1 (*PLK1*), and threonine tyrosine kinase (*TTK*)—are currently being evaluated as therapeutic targets in clinical trials for solid tumors (NCT04745689 and NCT05271292 for *AURKB*; NCT05983523 and NCT05827614 for *CHEK1*; NCT05450965 and NCT05358379 for *PLK1*; and NCT05251714 for *TTK*; Fig. 1C and D; Supplementary Fig. S1A). We focused on *CHEK1* because CHK1 inhibition has shown therapeutic potential in HGSOC, which is characterized by frequent *TP53* mutations, as is GIS-NEC (21). Among the 34 NEC cases, pancreatic NEC was the most prevalent (*n* = 12), followed by gastric NEC (*n* = 11; Supplementary Fig. S1B; Supplementary Table S6). Matched tumor and adjacent normal tissues were available in five GIS-NEC cases. All cases showed significantly higher *CHEK1* expression in tumors (Fig. 1E). Transcription factor subtypes differed between pancreatic and nonpancreatic NECs, and *CHEK1* expression also varied across these subtypes (Supplementary Fig. S1C; Supplementary Tables S6 and S7). NEC is histologically classified as small cell NEC (SCNEC) and large cell NEC (LCNEC) types, with SCNEC associated with significantly poorer prognosis (35). *CHEK1* expression was significantly higher in SCNEC than in LCNEC (Fig. 1F). SCNEC was also enriched in the *CHEK1*-high group (10/17 cases) compared with the *CHEK1*-low group (4/17 cases; Supplementary Table S6). No significant differences were observed in other clinicopathologic characteristics, including age, sex, race, smoking status, and alcohol consumption, between the *CHEK1*-high and *CHEK1*-low groups (Supplementary Table S6). These findings suggest that CHK1 is a potential therapeutic target in NEC, particularly in the more aggressive SCNEC.

**Figure 2.**
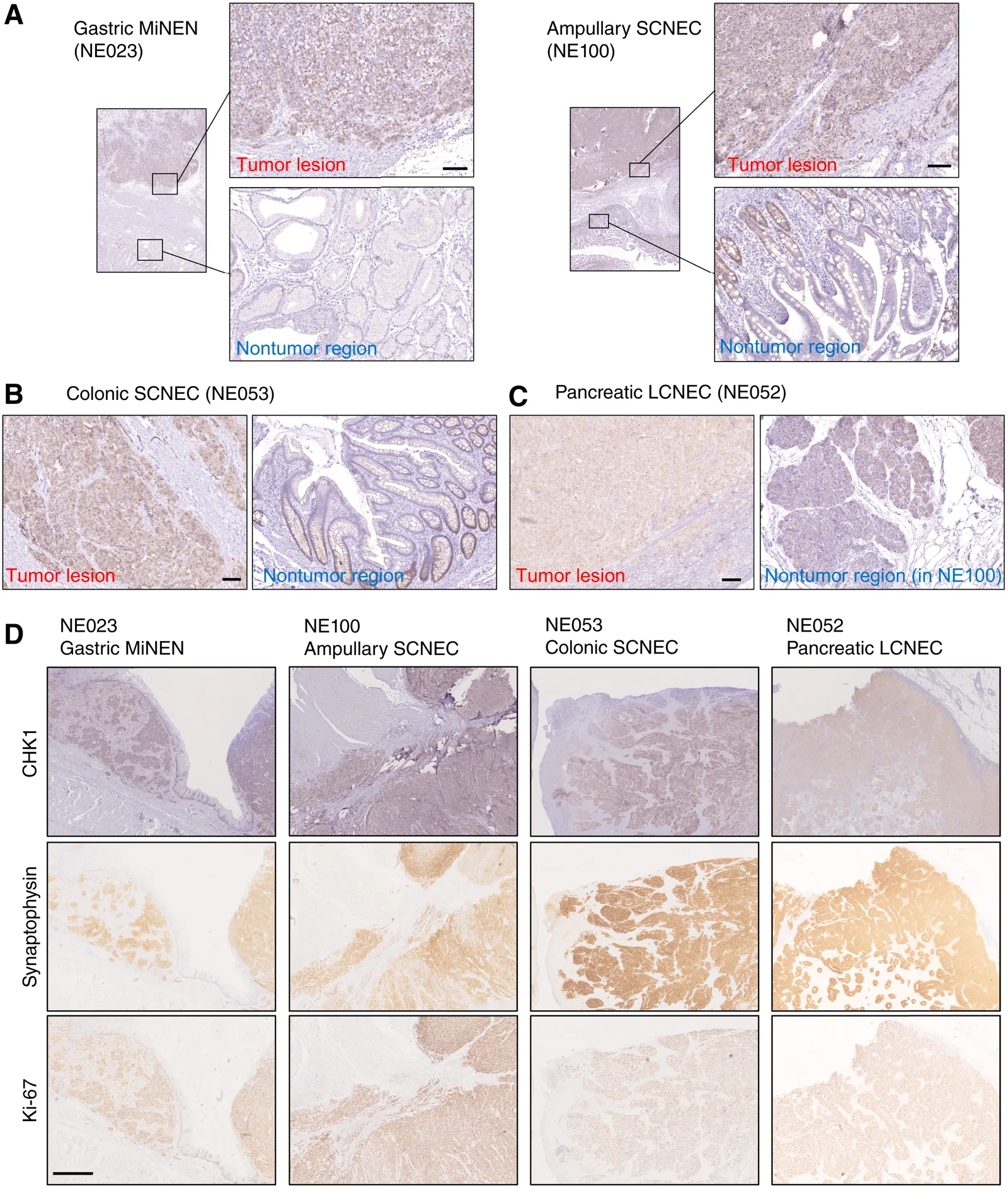
CHK1 expression in human GIS-NEC specimens. **A–C,** GIS-NEC tissues were stained with an anti-CHK1 antibody and hematoxylin. **D,** GIS-NEC tissues were stained with an anti-CHK1, anti-synaptophysin, or anti–Ki-67 antibody and hematoxylin. Scale bars, 100 μm. MiNEN, mixed neuroendocrine–non-neuroendocrine neoplasm.

Next, CHK1 protein expression was evaluated by IHC in four NEC cases of gastric, ampullary, colonic, and pancreatic origin (Fig. 2A–C). In the gastric and ampullary NECs, tumor and adjacent nontumorous tissues were present on the same sections, allowing direct comparison; in both cases, CHK1 staining was markedly stronger in the tumors than in the adjacent nontumorous tissues (Fig. 2A). Increased tumor staining was also observed in the colonic NEC (Fig. 2B). In the nontumorous intestinal mucosa, CHK1 staining was mainly observed at the base of the mucosal epithelium, consistent with the previously reported expression pattern of CHK1 in normal intestinal mucosa (Fig. 2A and B; refs. 36, 37). In the pancreatic NEC, although the nontumorous pancreatic tissue was obtained from a different case (an ampullary NEC case), CHK1 staining was likewise stronger in the tumor than in the nontumorous pancreatic tissue (Fig. 2C). In all four cases, CHK1-positive areas largely overlapped with synaptophysin- and Ki-67–positive areas (Fig. 2D). These findings provide histologic evidence of increased CHK1 protein expression in GIS-NEC.

### *CHEK1* expression correlates with poor prognosis and replication stress signaling in pancreatic NEC

**Figure 3.**
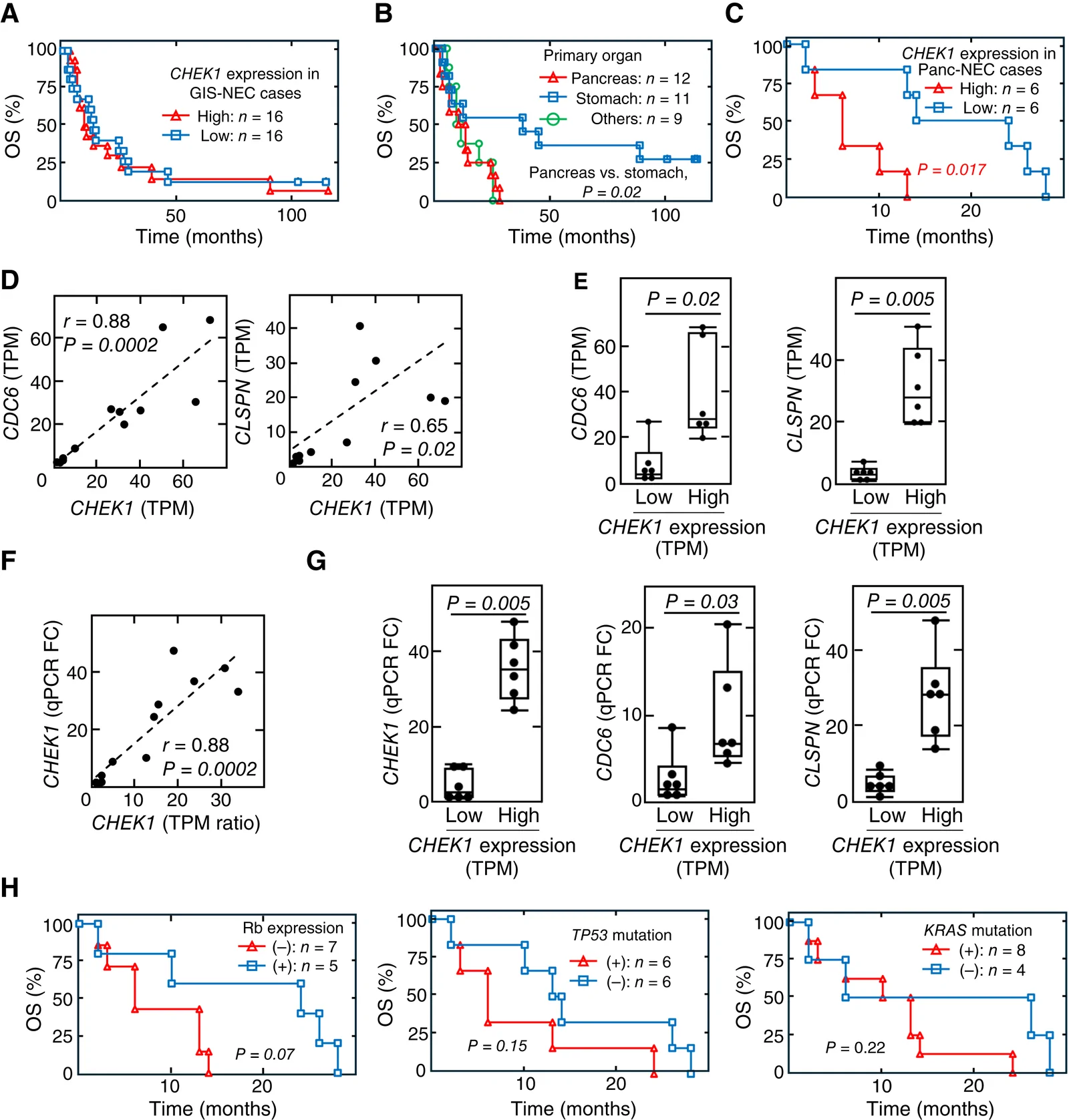
High *CHEK1* expression is associated with poor prognosis in pancreatic NEC. **A** and **C,** Relationship between *CHEK1* expression and OS in cases with GIS-NEC (**A**) and pancreatic NEC (**C**). **B,** OS stratified by primary tumor site in GIS-NEC. **D,** Scatter plots showing the correlation between *CHEK1* (*X*-axis) and *CDC6* (*Y*-axis; left) and *CHEK1* (*X*-axis) and *CLSPN* (*Y*-axis; right) in pancreatic NEC cases. The dotted black line represents the linear fit. **E,** Comparison of *CDC6* (left) and *CLSPN* (right) mRNA expression between *CHEK1*-low and *CHEK1*-high groups in pancreatic NEC cases. **F,** Scatter plots showing the correlation between *CHEK1* expression measured by RNA-seq (*X*-axis) and by qPCR (*Y*-axis). RNA-seq expression is shown as the ratio relative to the lowest expressing case, and qPCR expression is presented as FC normalized to *ACTB*. The dotted black line indicates the linear fit. **G,** Comparison of *CHEK1* (left), *CDC6* (center), and *CLSPN* (right) mRNA expression, measured by qPCR, between *CHEK1*-low and *CHEK1*-high groups defined by RNA-seq in pancreatic NEC cases. **H,** Relationship between OS and Rb expression (left), *TP53* mutation status (center), and *KRAS* mutation status (right) in pancreatic NEC cases. The log-rank test was used for statistical analysis (**A–C** and **H**). Pearson correlation coefficient (*r*) and the corresponding *P* value are indicated on each plot (**D** and **F**). Results are presented as box and whisker plots, in which the median is indicated by a line, the box represents the 25th to 75th percentiles, and the whiskers show the 5th to 95th percentiles (**E** and **G**). *P* values were calculated using the Mann–Whitney U test (**E** and **G**).

To evaluate the clinical relevance of *CHEK1* in GIS-NEC, we analyzed the association between *CHEK1* expression and prognosis. No significant association was observed between *CHEK1* expression and survival in the overall GIS-NEC cohort (Fig. 3A). Given the marked organ-specific biological heterogeneity of GIS-NEC (38), we next conducted subgroup analyses in pancreatic and gastric NECs, the two largest subgroups in our cohort. Patients with pancreatic NEC exhibited significantly poorer survival than those with gastric NEC (Fig. 3B). Whereas *CHEK1* expression was not associated with survival in gastric NEC, it was significantly associated with poorer prognosis in pancreatic NEC (Fig. 3C; Supplementary Fig. S2A), as previously reported in SCLC (39). We next examined the clinicopathologic characteristics of pancreatic NEC cases according to *CHEK1* expression (Supplementary Tables S7 and S8). The median age was 63 years (range, 53–79), comparable with that reported in a previous Surveillance, Epidemiology, and End Results Program (SEER)–based study of pancreatic NEC (40). Age, sex, smoking history, and alcohol consumption were similarly distributed between the *CHEK1*-high and *CHEK1*-low groups (Supplementary Tables S7 and S8). Numerically, the *CHEK1*-high group was enriched for *ASCL1* and SCNEC, whereas *PTF1A* and *YAP1* subtypes were observed only in the *CHEK1*-low group; however, the limited sample size precluded definitive conclusions.

To determine whether *CHEK1* expression is linked to replication stress pathways, we examined correlations between *CHEK1* expression and the replication stress–related genes cell division cycle 6 (*CDC6*) and claspin (*CLSPN*). In pancreatic NEC, *CHEK1* expression correlated significantly with both *CDC6* and *CLSPN* (Fig. 3D), suggesting increased reliance on replication stress signaling. Accordingly, the high *CHEK1* expression group showed significantly higher expression of *CDC6* and *CLSPN* than the *CHE*K1-low group (Fig. 3E). Moreover, high expression of *CDC6* and *CLSPN* was also associated with significantly poorer prognosis in pancreatic NEC (Supplementary Fig. S2B). Notably, all *CLSPN*-high cases overlapped with *CHEK1*-high cases, whereas five of six *CDC6*-high cases were also classified as *CHEK1* high, further supporting the link between *CHEK1* expression and replication stress signaling in pancreatic NEC (Supplementary Table S7). In contrast, in gastric NEC, *CHEK1* expression correlated with *CDC6* but not with *CLSPN* (Supplementary Fig. S2C), and no significant difference in *CDC6* or *CLSPN* expression was observed between the high- and low-*CHEK1* groups (Supplementary Fig. S2D). These findings further support that replication stress signaling is more prominently activated in pancreatic NEC than in gastric NEC.

To validate *CHEK1* expression in pancreatic NEC, we performed quantitative PCR (qPCR). As appropriate reference genes differ among cancer types, candidate reference genes were evaluated based on RNA-seq data from 12 pancreatic NEC cases (41, 42). *ACTB*, which showed high TPM values and low expression variability (CV), was selected as the reference gene (Supplementary Fig. S2E and S2F). *CHEK1* expression levels were calculated as fold changes (FC) relative to the lowest expressing sample (case NE101), and RNA-seq TPM values were normalized in the same manner to generate ratios. qPCR FCs showed a strong correlation with RNA-seq TPM ratios (Pearson *r* = 0.88; Fig. 3F), confirming the reliability of the RNA-seq measurements. Similar correlations were also observed for *CDC6* and *CLSPN* (Supplementary Fig. S2G). Furthermore, cases with high *CHEK1* expression based on TPM values also showed significantly higher expression of *CHEK1*, *CDC6*, and *CLSPN* by qPCR (Fig. 3G). Together, these concordant findings obtained using two independent methods provide orthogonal validation of the gene expression data.

Notably, in our pancreatic NEC cohort, Rb expression status and the presence of *TP53* or *KRAS* mutation were not significantly associated with patient survival (Fig. 3H; Supplementary Fig. S2H; Supplementary Table S7), suggesting that *CHEK1* expression may represent a prognostic indicator in addition to these canonical alterations.

### Intratumoral heterogeneity and clonal structure of *CHEK1*-associated replication stress signaling

**Figure 4.**
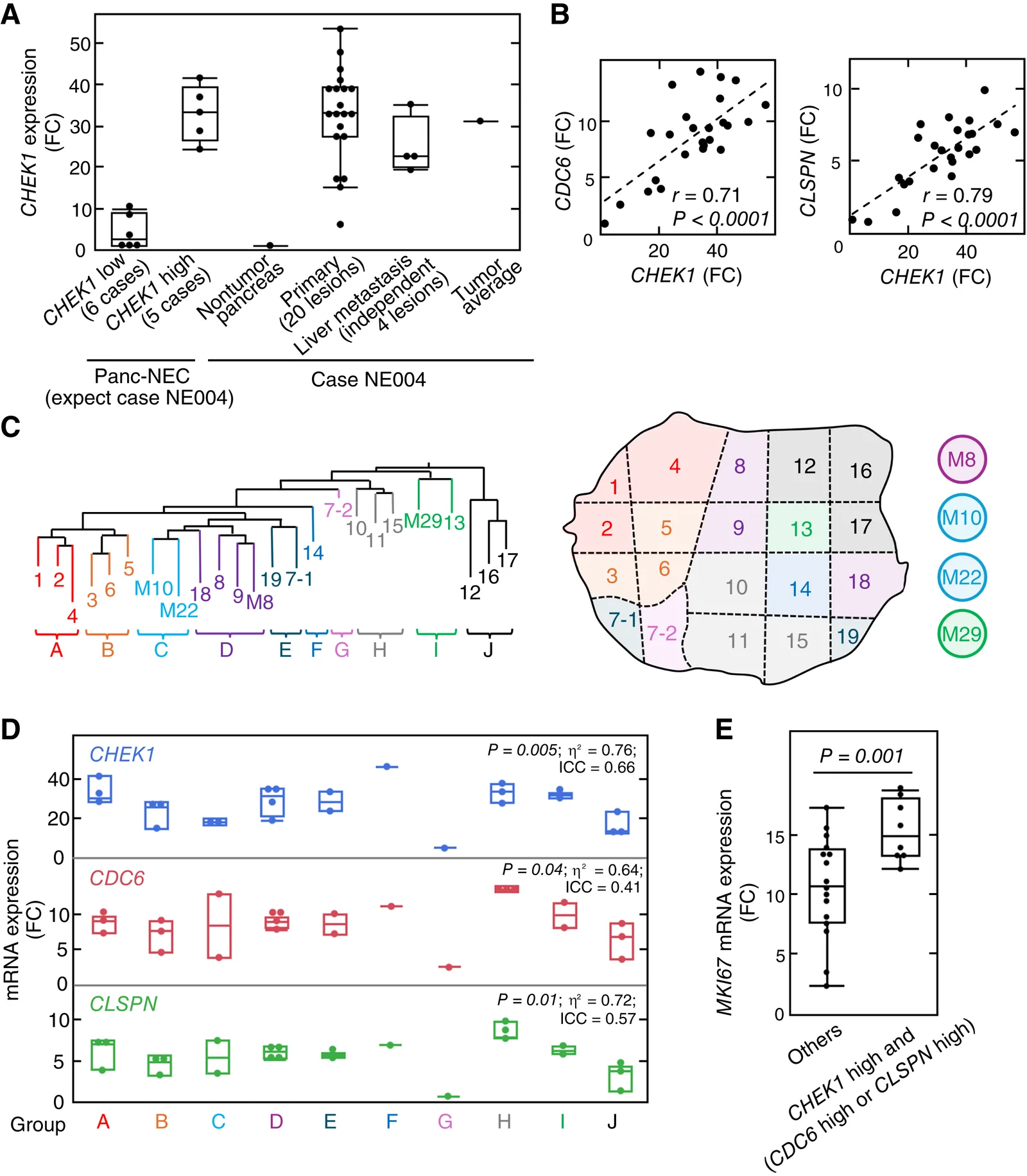
Spatial co-localization of *CHEK1*, *CDC6*, and *CLSPN* expression within pancreatic NEC (Panc-NEC) tumors. **A,***CHEK1* expression levels were measured by qPCR and normalized to *ACTB*. FC is shown for 11 GIS-NEC cases and 24 tumor regions from case NE004. **B,** Scatter plots showing the correlation between *CHEK1* (*X*-axis) and *CDC6* (*Y*-axis; left) and *CHEK1* (*X*-axis) and *CLSPN* (*Y*-axis; right) in 24 tumor regions from case NE004. The dotted black line represents the linear fit. Pearson correlation coefficient (r) and the corresponding *P* value indicated. **C,** Phylogenetic tree of case NE004 tumor regions was taken directly from Yachida and colleagues (13), in which it was inferred using RAxML based on mutation and LOH patterns from exome sequencing data. Tumor regions were classified into 10 groups (A–J) according to the phylogenetic tree (left), and each region is illustrated with colors indicating the predicted subclones (right). **D,** Expression levels of *CHEK1* (top), *CDC6* (middle), and *CLSPN* (bottom) were measured by qPCR, normalized to *ACTB*, and presented as FC relative to the adjusted normal pancreas. Tumor regions were grouped A–J shown in Fig. 3C. **E,** Expression level of *MKI67* was measured by qPCR, normalized to *ACTB*, and presented as FC relative to the adjusted normal pancreas, stratified according to *CHEK1*, *CDC6*, and *CLSPN* expression levels. Results are presented as box and whisker plots, in which the median is indicated by a line, the box represents the 25th to 75th percentiles, and the whiskers show the 5th to 95th percentiles (**A**, **D**, and **E**). Differences across regions were assessed by one-way ANOVA (*P* and effect size indicated), and intraregional variability was quantified by CV and ICC using a linear mixed-effects model (**D**).

To investigate the association between intratumoral *CHEK1* expression and replication stress signaling, we analyzed tumor specimens obtained from multiple spatially distinct regions within the largest section of the NE004 autopsy specimen, which was classified as *CHEK1* overexpressing in this study (Supplementary Table S7). In our previous study, we performed whole-exome sequencing across multiple regions of both the primary tumor and metastatic lesions from this patient to delineate the genomic evolutionary landscape (13). Using RNA derived from the same regions, we evaluated *CHEK1* and replication stress–related gene expression across tumor sites. *CHEK1* expression was elevated across all tumor regions compared with matched normal tissue, with a wide range (5.5- to 47.2-fold), indicating marked intratumoral heterogeneity (Fig. 4A). Twenty-three of 24 regions exhibited higher *CHEK1* expression than the six *CHEK1*-low cases, supporting the classification of NE004 as *CHEK1* high. Despite this heterogeneity, *CHEK1* expression showed strong positive correlations with *CDC6* and *CLSPN* across regions (Fig. 4B; Supplementary Fig. S3A), with no significant difference between primary and metastatic lesions.

Based on phylogenetic relationships reported in our previous study (13), the 24 regions were classified into 10 groups (A–J; Fig. 4C), with some groups containing a single region. Stratification by these groups revealed that *CHEK1*, *CDC6*, and *CLSPN* exhibited greater intergroup than intragroup variability, with significant differences across groups (*CHEK1*: *P* = 0.0047 and η^2^ = 0.755; *CDC6*: *P* = 0.0419 and η^2^ = 0.642; *CLSPN*: *P* = 0.010 and η^2^ = 0.722; Fig. 4D). Linear mixed-effects modeling demonstrated likewise moderate-to-high ICCs (ICC = 0.66, 0.41, and 0.57 for *CHEK1*, *CDC6*, and *CLSPN*, respectively), indicating that a substantial proportion of expression variability is attributable to phylogenetically defined group structure. To further validate this finding, the same analyses were performed for two commonly used reference genes peptidylprolyl isomerase A (*PPIA*) and ribosomal protein L13a (*RPL13A*) as negative controls and two additional replication stress–associated genes minichromosome maintenance complex component 2 (*MCM2*) and ribonucleotide reductase regulatory subunit M2 (*RRM2*). As expected, *PPIA* and *RPL13A* showed no significant variation among phylogenetic groups (*P* = 0.332 and 0.833, respectively) and exhibited low ICC values (0.09 and 0, respectively; Supplementary Fig. S3B). In contrast, *MCM2* and *RRM2* expression significantly correlated with *CHEK1* expression across the 24 sampled regions (*MCM2*: *r* = 0.78 and *P* < 0.0001; *RRM2*: *r* = 0.49 and *P* = 0.015; Supplementary Fig. S3C) and demonstrated similar phylogeny-associated expression patterns (*MCM2*: *P* = 0.0069, η^2^ = 0.739, and ICC = 0.60; *RRM2*: *P* = 0.044, η^2^ = 0.639, and ICC = 0.39). A summary comparison of *P* values, effect sizes (η^2^), and ICCs across all analyzed genes is provided in Supplementary Fig. S3D. Among all regions, the top 10 highest expressing regions for each gene showed significantly elevated *MKI67* expression (Supplementary Fig. S3E). Notably, 8 of the 10 regions with the highest *CHEK1* expression overlapped with the top 10 regions for *CDC6* or *CLSPN* expression, supporting coordinated upregulation (Supplementary Fig. S3F). Similarly, these overlapping regions also exhibited significantly higher *MKI67* expression compared with other regions (Fig. 3E). These findings indicate that replication stress signaling associated with *CHEK1* expression is spatially heterogeneous within the tumor and may partly reflect underlying phylogenetic relationships.

### CHK1 inhibitor effectively suppresses the proliferation of GIS-NEC cell lines

**Figure 5.**
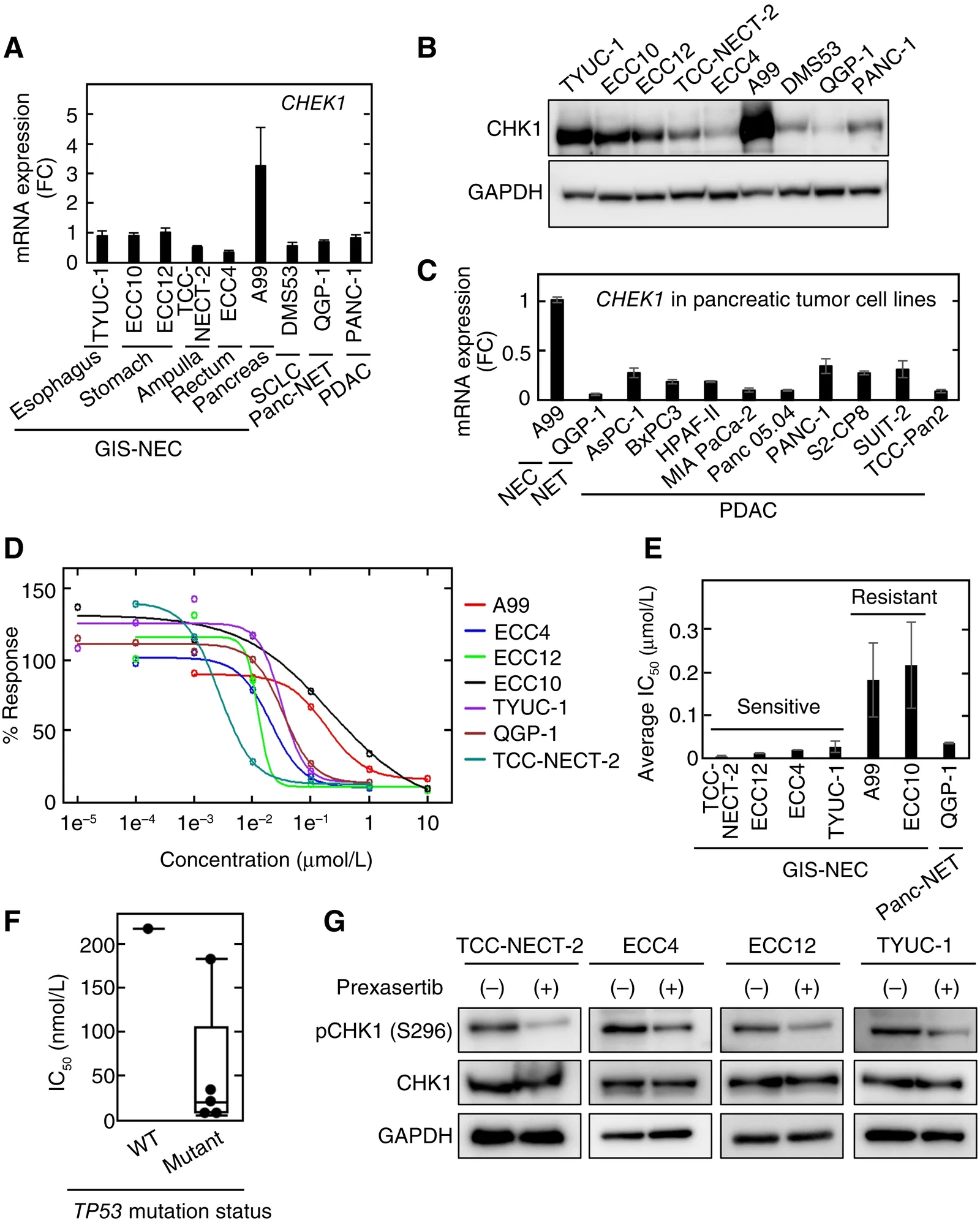
Assessment of CHK1 inhibitor sensitivity in GIS-NEC cell lines. **A** and **C,** Expression levels of *CHEK1* mRNA in GIS-NEC, SCLC, pancreatic NET, PDAC cell lines (**A**), and 11 pancreatic tumor cell lines (**C**) were measured by quantitative RT-PCR and normalized to *GAPDH*. Results are presented as FCs relative to TYUC-1 cells (**A**) and A99 cells (**C**) and are expressed as means ± SD from three independent experiments. **B,** Lysates from GIS-NEC, SCLC, pancreatic NET, and PDAC cell lines, as shown in Fig. 2A, were subjected to immunoblotting with anti-CHK1 and anti-GAPDH antibodies. GAPDH served as the loading control. **D,** Viability of GIS-NEC and pancreatic NET cells after prexasertib treatment. Data represent mean ± SD of two independent experiments, each with six replicate wells. **E,** IC_50_ values for prexasertib in the indicated cell lines. Data are shown as mean ± SD of two independent experiments. **F,** IC_50_ values for prexasertib in GIS-NEC cell lines, compared between *TP53*-mutant and *TP53* WT groups. Data are presented as box and whisker plots, in which the horizontal line indicates the median, the box represents the interquartile range (25th to 75th percentiles), and the whiskers denote the 5th to 95th percentiles. **G,** Four GIS-NEC cell lines were treated with prexasertib (at the concentration corresponding to the respective IC_50_) for 24 hours. Lysates were subjected to immunoblotting with the indicated antibodies. *GAPDH* was used as the loading control.

*CHEK1* expression was examined in six GIS-NEC cell lines, one SCLC cell line, one pancreatic NET cell line, and one PDAC cell line. All GIS-NEC cell lines harbored *TP53* or *RB1* mutation (30). RNA-seq and qPCR analyses revealed that the pancreatic NEC cell line A99 (24) derived from case NE024, classified as *CHEK1* high in this study (Supplementary Table S7), exhibited the highest *CHEK1* expression, followed by TYUC-1, ECC10, and ECC12 (Fig. 5A; Supplementary Fig. S4A). CHK1 protein levels generally followed the mRNA expression patterns (Fig. 5B). To confirm that high *CHEK1* expression is specific to pancreatic NEC rather than being broadly elevated across pancreatic tumors, we compared A99 cells with one pancreatic NET cell line and nine PDAC cell lines. The NET cell line QGP-1 and the PDAC cell line PANC-1 shown in Fig. 2A and B were included in this expanded panel. *CHEK1* expression in A99 cells was markedly higher than that in all pancreatic tumor cell lines at the mRNA level (Fig. 5C), suggesting that *CHEK1* overexpression may be a characteristic feature of pancreatic NEC cell line A99.

We next assessed the sensitivity of these cell lines to the CHK1 inhibitor prexasertib by determining IC_50_ values. Four GIS-NEC cell lines (TYUC-1, ECC12, TCC-NECT-2, and ECC4) exhibited IC_50_ values below 50 nmol/L, comparable with the CHK1 inhibitor–sensitive SCLC cell line DMS53 (Fig. 5D and E; Supplementary Fig. S4B; ref. 19). In contrast, A99 and ECC10 cells showed IC_50_ values exceeding 100 nmol/L, comparable with that of the CHK1 inhibitor–resistant PDAC cell line PANC-1 (Fig. 5D and E; Supplementary Fig. S4B; ref. 43). Accordingly, these four cell lines were defined as CHK1 inhibitor sensitive, whereas A99 and ECC10 were classified as resistant. Expression of key transcriptional regulators varied across the cell lines (Supplementary Fig. S4C). Given the reported association between *TP53* deficiency and CHK1 inhibitor sensitivity, we also examined *TP53* status in the six GIS-NEC cell lines. Five cell lines harbored *TP53* alterations, whereas ECC10 retained wild-type (WT) TP53 (Supplementary Table S3; refs. 24, 30). Four of the five *TP53*-mutant cell lines (TCC-NECT-2, ECC12, ECC4, and TYUC-1) had relatively low IC_50_ values for prexasertib, whereas the *TP53*-mutant A99 cell line showed resistance comparable with that of *TP53* WT ECC10 (Fig. 5E and F). Although *TP53* alterations may contribute to CHK1 inhibitor sensitivity, *TP53* status alone does not explain the differential response to prexasertib among the GIS-NEC cell lines examined. Furthermore, prexasertib sensitivity did not correlate with *CHEK1* expression levels or transcriptional regulator patterns, suggesting that additional determinants influence the response to CHK1 inhibition. In CHK1 inhibitor–sensitive cell lines, CHK1 phosphorylation was effectively suppressed at concentrations below 50 nmol/L, in line with their respective IC_50_ values (Fig. 5G).

### CHK1 inhibition induces replication stress and suppresses tumor growth in GIS-NEC

**Figure 6.**
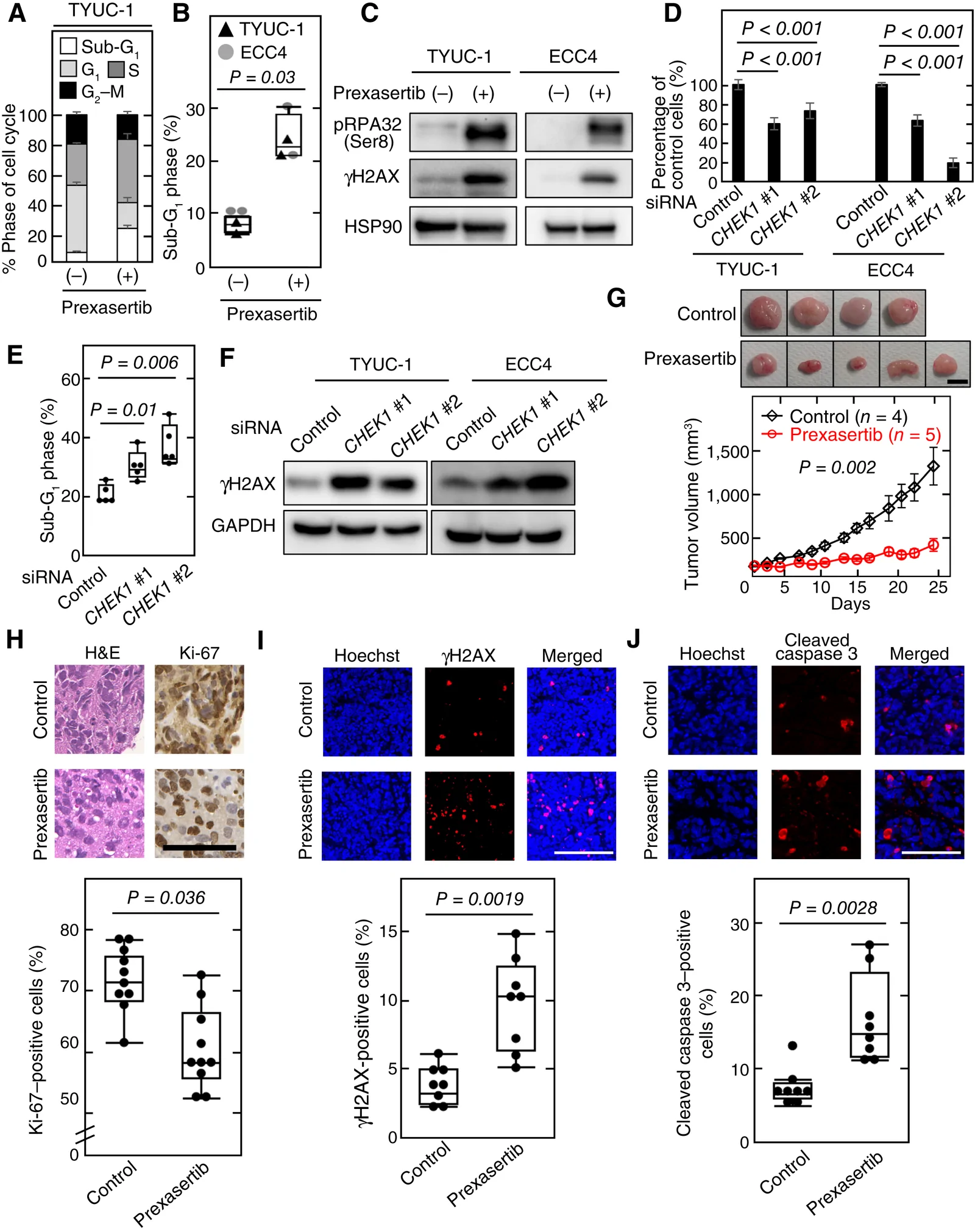
Effects of CHK1 inhibition in sensitive GIS-NEC cell lines. **A,** Cell-cycle analysis of TYUC-1 cells treated with prexasertib at the IC_50_ concentration (*n* = 2). Flow cytometry was performed 24 hours after treatment. The percentage of cells in each cell-cycle phase is shown as means ± SD. **B,** Cell-cycle analysis data from independent experiments using TYUC-1 and ECC4 cells shown in Fig. 5A and Supplementary Fig. S5B were combined. The percentages of cells in the sub-G_1_ phase are shown as box and whisker plots. **C,** TYUC-1 and ECC4 cells were treated with prexasertib at the respective IC_50_ concentration for 24 hours. Protein lysates were probed by immunoblotting with the indicated antibodies. HSP90 was used as the loading control. **D,** Viability assays were performed in TYUC-1 and ECC4 cells transfected with control or *CHEK1* siRNA. Measurements were taken 72 hours (TYUC-1) and 120 hours (ECC4) after transfection. Results are presented as means ± SD from three independent experiments. **E,** Cell-cycle analysis data from independent experiments using the TYUC-1 and ECC4 cells shown in Supplementary Fig. S5E and S5F were combined. The percentages of cells in the sub-G_1_ phase were plotted using box and whisker plots. **F,** Two GIS-NEC cell lines were transfected with 25 nmol/L control or CHEK1 siRNA. Protein lysates were collected 72 hours (TYUC-1) and 120 hours (ECC4) after transfection and probed with anti-γH2AX and anti-GAPDH antibodies. GAPDH served as the loading control. **G,** TYUC-1 cells were subcutaneously implanted into immunodeficient mice. Prexasertib (40 mg/kg/week, *n* = 5) or vehicle control (*n* = 4) was administered via intraperitoneal injection twice weekly. Top, images of extirpated xenografts; bottom, images of tumor volume changes over time. Data are expressed as means ± SD. **H,** Sections prepared from TYUC-1 xenograft tumors were stained with hematoxylin and anti–Ki-67 antibody. Top, representative images. The percentage of Ki-67–positive cells relative to the total number of cells per field (*n* = 10 fields) is quantified. H&E, hematoxylin and eosin. **I** and **J,** Sections prepared from TYUC-1 xenograft tumors were stained with a nuclear dye (blue) and either anti-γH2AX antibody (red; **I**) or anti–cleaved caspase 3 antibody (red; **J**). Top, representative images. The percentages of γH2AX-positive cells (**I**) and cleaved caspase 3–positive cells (**J**) relative to the total number of cells per field (*n* = 8 fields). Data are presented as box and whisker plots, in which the horizontal line indicates the median, the box represents the interquartile range (25th to 75th percentiles), and the whiskers denote the 5th to 95th percentiles (**B**, **E**, and **H–J**). Statistical significance was determined using the Mann–Whitney U test (**B**, **D**, **E**, **G**, and **H–J**). Scale bars, 10 mm (**G**) and 100 μm (**H–J**).

Prexasertib has been reported to alter the cell-cycle distribution in various tumor types (19, 43, 44). To evaluate this effect in GIS-NEC, we performed cell-cycle analysis in two prexasertib-sensitive NEC cell lines, TYUC-1 and ECC4. TYUC-1 cells, which exhibited the highest IC_50_ value among the sensitive lines, were included to assess effects in a relatively less sensitive context, and ECC4 was selected as a representative model of colorectal NEC, the most common primary site in GIS-NEC (38). Prexasertib was administered at concentrations corresponding to the IC_50_ value of each cell line. Combined analysis of TYUC-1 and ECC4 cells showed a significant increase in the sub-G_1_ population following treatment in both cell lines, whereas G_1_- and S-phase fractions were unchanged, likely reflecting baseline differences between cell lines (Fig. 6A and B; Supplementary Fig. S5A–S5C). Prexasertib treatment markedly increased phosphorylated RPA32 (pRPA32) and γH2AX levels (Fig. 6C), indicating induction of replication stress and DNA damage. *CHEK1* knockdown using two independent siRNAs recapitulated these effects, suppressing proliferation, increasing the sub-G_1_ fraction, and elevating γH2AX levels (Fig. 6D–F; Supplementary Fig. S5D–S5F).

We next evaluated the antitumor efficacy of prexasertib *in vivo* using TYUC-1 xenografts. Prexasertib significantly suppressed tumor growth compared with the control (Fig. 6G). IHC analysis showed reduced Ki-67 positivity and increased γH2AX- and cleaved caspase 3–positive cells (Fig. 6H–J), indicating decreased proliferation and increased DNA damage–associated apoptosis.

### WNT inhibition enhances sensitivity to CHK1 inhibition

**Figure 7.**
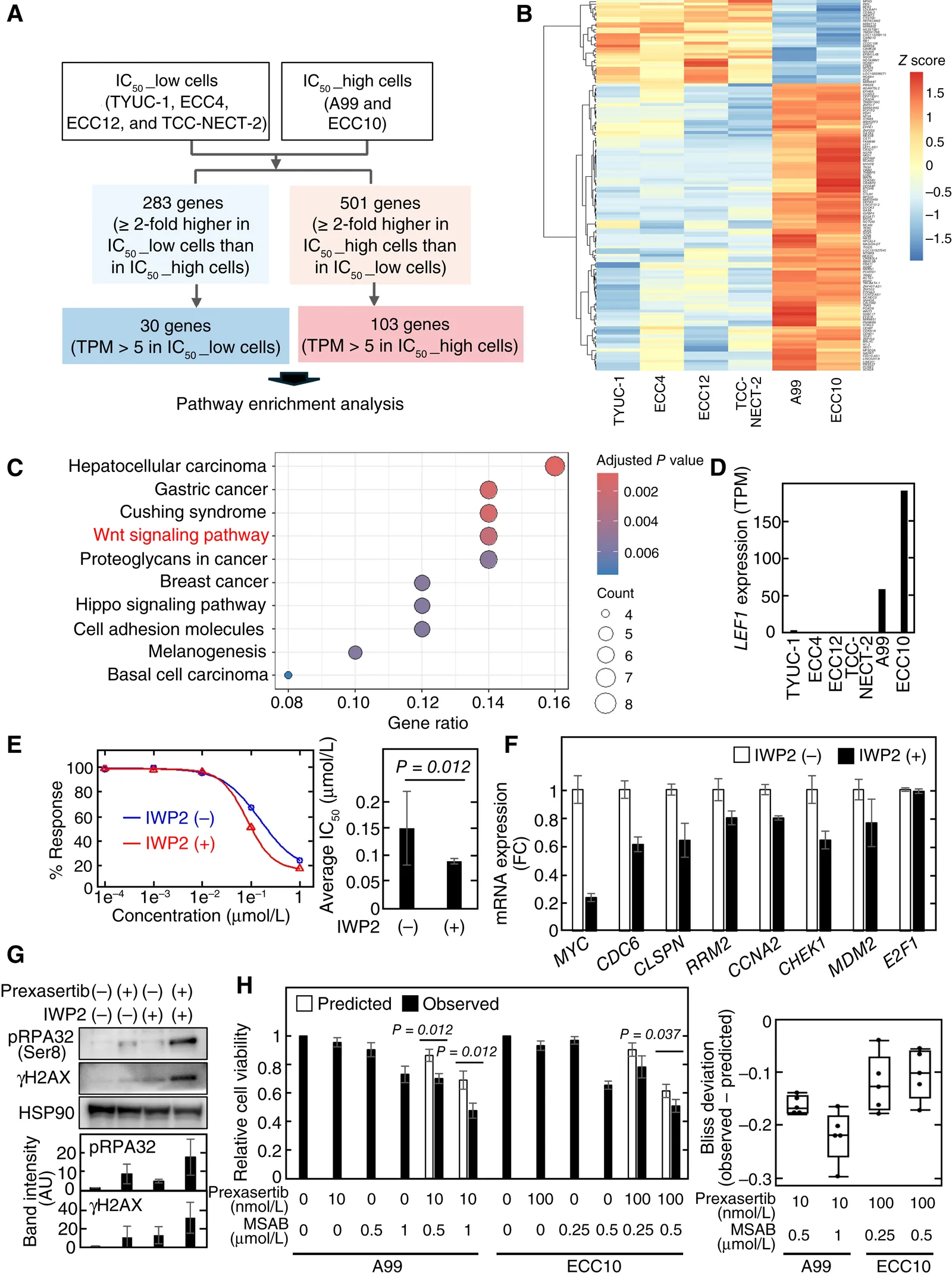
Correlation between expression of WNT signaling–related genes and reduced sensitivity to CHK1 inhibitor. **A,** Flowchart illustrating the gene selection process for pathway enrichment analysis. Genes with ≥2-fold expression differences between IC_50__high and IC_50__low cells were first identified. From these, genes with TPM >5 in IC_50__high or IC_50__low cells (depending on the direction of overexpression) were extracted for KEGG pathway analysis. **B,** Heatmap depicting the expression of 133 genes extracted in Fig. 6A that show ≥2-fold differences between IC_50__high and IC_50__low cells and have TPM >5 in either group. **C,** Pathway enrichment analysis of the 133 genes shown in Fig. 6A and B using the KEGG database. **D,** Expression levels of *LEF1* mRNA in six GIS-NEC cell lines were measured by RNA-seq. **E,** Viability of A99 cells after prexasertib treatment ± 10 μmol/L IWP2. Left, data represent mean ± SD of three independent experiments, each with six replicate wells; right, IC_50_ values are shown as mean ± SD of three independent experiments. **F,** Expression levels of the indicated genes in A99 cells treated with ± 10 μmol/L IWP2 for 48 hours were measured by quantitative RT-PCR and normalized to *GAPDH*. Data are shown as FC relative to the untreated condition and represent mean ± SD of three independent experiments. **G,** A99 cells were treated with 50 nmol/L prexasertib (24 hours), 10 μmol/L IWP2 (48 hours), or both. Lysates were analyzed by immunoblotting with the indicated antibodies. Top, HSP90 was used as the loading control. Band intensities of pRPA32 (middle) and γH2AX (bottom) were quantified using ImageJ, normalized to HSP90, and expressed as arbitrary units (AU), mean ± SD of three independent experiments relative to untreated cells. **H,** Cell viability following treatment with prexasertib and MSAB, expressed relative to that of untreated cells. Left, white bars indicate the cell viability predicted under the assumption of Bliss independence, whereas black bars indicate the observed cell viability. Data are shown as FC relative to the untreated condition and represent the mean ± SD of five independent experiments. Right, the Bliss deviation, calculated as observed minus predicted cell viability. Data are presented as box and whisker plots, with the horizontal line indicating the median, the box representing the interquartile range (25th–75th percentiles), and the whiskers denoting the 5th–95th percentiles. Statistical significance was determined using the Mann–Whitney U test (**E** and **H**).

To identify molecular determinants of sensitivity to CHK1 inhibition, we compared gene expression profiles between prexasertib-sensitive (IC_50__low) and -resistant (IC_50__high) GIS-NEC cell lines. Previously reported determinants of CHK1 inhibitor sensitivity, including genes involved in DNA replication, cell-cycle regulation, and checkpoint signaling (22, 44–47), were not associated with prexasertib IC_50_ values in GIS-NEC (Supplementary Fig. S6A). We therefore analyzed the RNA-seq data to identify genes differentially expressed between prexasertib-sensitive and -resistant cell lines and identified 133 genes with ≥2-fold differential expression between the two groups (Fig. 7A and B). KEGG pathway analysis revealed significant enrichment of the WNT signaling pathway (Fig. 7C). *LEF1*, a key β-catenin–dependent transcription factor, together with WNT ligands (*WNT3* and *WNT6*) and *FRAT1*, a WNT signaling activator that stabilizes β-catenin via inhibition of GSK-3β (48–50), was markedly upregulated in the resistant cell lines (Fig. 7D; Supplementary Fig. S6B). Although canonical WNT target genes such as *AXIN2* were not prominently upregulated, these findings suggest a potential involvement of WNT-related signaling in resistance.

To assess functional relevance, we inhibited WNT signaling using IWP2, which suppressed *AXIN2* expression in A99 cells but not in *APC*-mutant ECC10 cells, indicating constitutive pathway activation in the latter (Supplementary Fig. S6C). We therefore focused subsequent analyses on A99 cells. IWP2 reduced the prexasertib IC_50_ in A99 cells by approximately 40% (151.3–89.1 nmol/L; Fig. 7E). Mechanistically, WNT inhibition reduced *MYC* expression and suppressed *E2F* target genes involved in DNA replication and checkpoint control—including *CDC6*, *CLSPN*, *RRM2*, *CCNA2*, and *CHEK1*—despite no apparent change in *E2F1* levels. Among these, *CDC6* showed the greatest reduction, suggesting impaired replication origin licensing, a process that prepares replication origins for subsequent DNA synthesis and is essential for the proper initiation of DNA replication (Fig. 7F). *MDM2* expression was also modestly decreased, supporting suppression of *MYC*–*E2F*–dependent transcriptional programs (Fig. 7F). Combined treatment with IWP2 and low-dose prexasertib (50 nmol/L) further increased γH2AX and pRPA32 levels, indicating enhanced replication stress and DNA damage (Fig. 7G), and more strongly suppressed *CDC6* expression compared with either treatment alone (Supplementary Fig. S6D). Next, we used methyl 3-{[(4-methylphenyl)sulfonyl]amino}benzoate (MSAB) to further assess whether pharmacologic inhibition of WNT–β-catenin signaling affects prexasertib sensitivity in A99 and ECC10 cells. MSAB suppressed *MYC* expression in both cell lines, confirming its expected effect on a downstream target of WNT–β-catenin signaling (Supplementary Fig. S6E). The IC_50_ values of MSAB were 3.2 and 0.9 μmol/L in A99 and ECC10 cells, respectively (Supplementary Fig. S6F). In both cell lines, MSAB treatment reduced the prexasertib IC_50_ relative to that in the absence of MSAB, based on values obtained from separate experiments (43.7 vs. 182.5 nmol/L in A99 cells and 121.3 vs. 216.5 nmol/L in ECC10 cells, respectively; Fig. 5E; Supplementary Fig. S6G). We further assessed the combined effects of prexasertib and MSAB using Bliss independence analysis. In both cell lines, observed cell viability was significantly lower than the Bliss-predicted viability under the combination conditions tested (prexasertib 10 nmol/L with MSAB at 0.5 or 1 μmol/L in A99 cells; *P* = 0.012 and prexasertib 100 nmol/L with MSAB at 0.5 μmol/L in ECC10 cells; *P* = 0.037; Fig. 7H). The Bliss deviation (observed viability − Bliss-predicted viability) was −0.22 (range, −0.30 to −0.17) in A99 cells treated with prexasertib 10 nmol/L and MSAB 1 μmol/L and −0.10 (range, −0.17 to −0.05) in ECC10 cells treated with prexasertib 100 nmol/L and MSAB 0.5 μmol/L (Fig. 7H). These results indicate that pharmacologic inhibition of WNT–β-catenin signaling enhances prexasertib sensitivity and supports a synergistic interaction between CHK1 and WNT–β-catenin pathway inhibition in both prexasertib-resistant cell lines under the tested condition. WNT inhibition may promote a replication-compromised state through suppression of *MYC*–*E2F*–driven programs, thereby increasing reliance on CHK1-mediated checkpoint control and enhancing sensitivity to CHK1 inhibition.

### Identification and clinical relevance of LRP8 expression in pancreatic NEC

**Figure 8.**
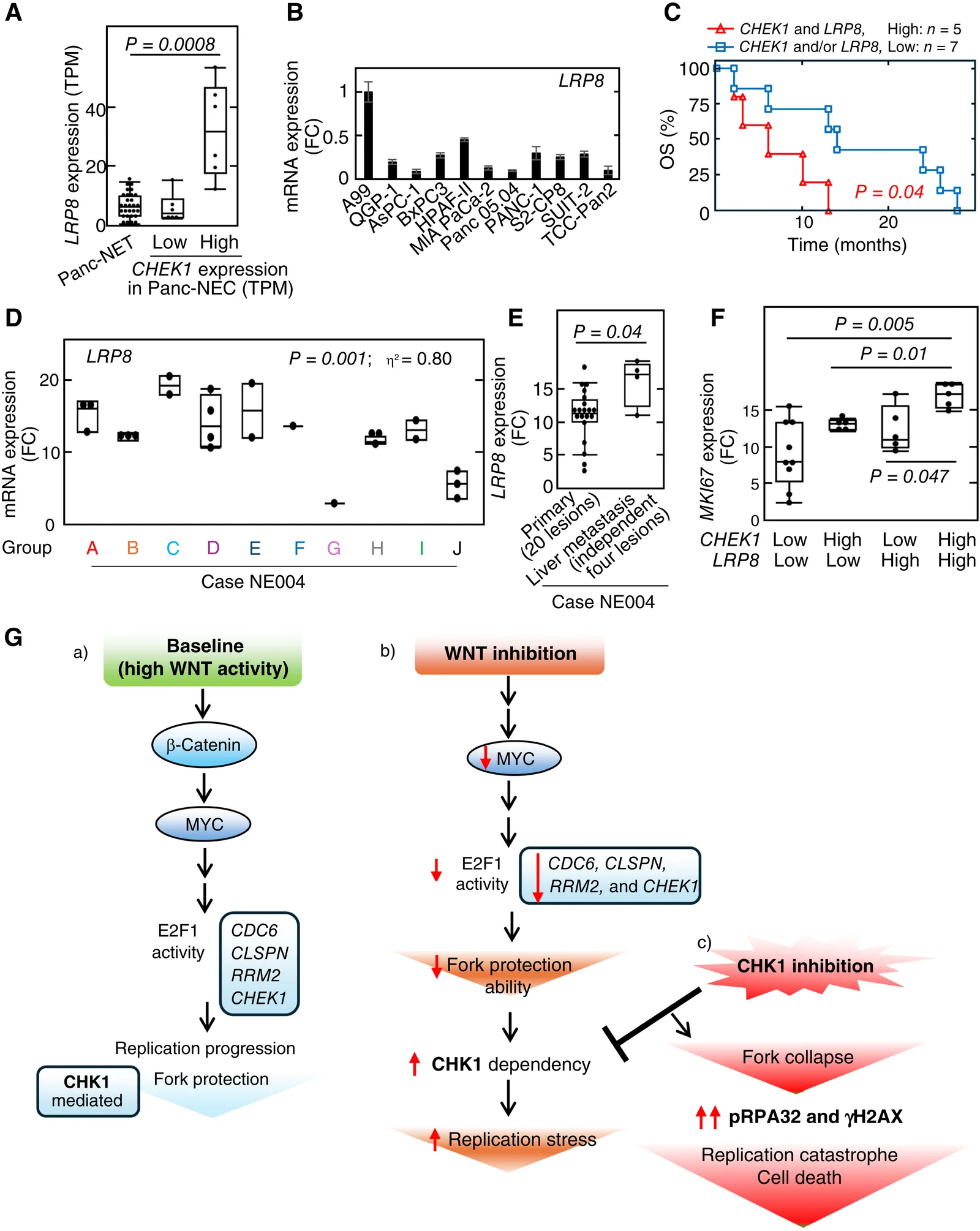
*LRP8* expression in pancreatic NEC (Panc-NET) and association with *CHEK1* and clinical aggressiveness. **A,** Comparison of *LRP8* mRNA expression between pancreatic NET case and *CHEK1*-low and *CHEK1*-high groups in pancreatic NEC cases. **B,** Expression levels of *LRP8* mRNA in 11 pancreatic tumor cell lines were measured by quantitative RT-PCR and normalized to *GAPDH*. Results are presented as FCs relative to A99 cells and are expressed as means ± SD from three independent experiments. **C,** OS of pancreatic NEC cases comparing both *CHEK1*- and *LRP8*-high groups with others. **D,** Expression level of *LRP8* was measured by qPCR, normalized to *ACTB*, and shown as FC relative to adjusted normal pancreas. Tumor regions are grouped A–J as in Fig. 3C. **E,** Expression level of *LRP8* was measured by qPCR, normalized to *ACTB*, comparing primary lesions and liver metastases. **F,** Expression level of *MKI67* was measured by qPCR, normalized to *ACTB*, and presented as FC relative to the adjusted normal pancreas, stratified according to *CHEK1* and *LRP8* expression levels. **G,** Schematic of signaling: (a) baseline high WNT activity, (b) WNT inhibition, and (c) combined WNT and CHK1 inhibition. Results are presented as box and whisker plots, in which the median is indicated by a line, the box represents the 25th to 75th percentiles, and the whiskers show the 5th to 95th percentiles (**A**, **D**, and **E**). Differences across regions were assessed by one-way ANOVA (*P* and effect size indicated), and intraregional variability was quantified by CV and ICC using a linear mixed-effects model (**D**). *P* values were calculated using the Kruskal–Wallis (**A**) and Mann–Whitney U tests (**E**). The log-rank test was used for statistical analysis (**C**).

As WNT inhibition enhanced sensitivity to CHK1 inhibition in pancreatic NEC cell line A99, we next examined the expression of WNT signaling–related genes in pancreatic NEC. Comparison between *CHEK1*-high and *CHEK1*-low cases revealed significant upregulation of several WNT-related genes, including *LRP4*, *LRP8*, and *FZD8*, in *CHEK1*-high cases (Fig. 8A; Supplementary Fig. S7A). RNA-seq analysis showed that *LRP4* and *LRP8* were highly expressed in A99 cells, whereas *FZD8* was not (Supplementary Fig. S7B). Across 11 pancreatic tumor cell lines, *LRP4* and *LRP8* expression was enriched in A99 cells, whereas *FZD8* expression was more broadly distributed (Fig. 8B; Supplementary Fig. S7C). *LRP8* has been reported to function as a positive regulator and *LRP4* as a negative regulator of WNT signaling (51, 52). We next assessed their clinical relevance in pancreatic NEC. High *LRP8* expression was significantly associated with poorer survival (*P* = 0.007), whereas *LRP4* expression was not (Supplementary Fig. S7D). Furthermore, patients with concurrent high *CHEK1* and *LRP8* expression showed worse survival than other groups (*P* = 0.04; Fig. 8C), suggesting that *LRP8*-associated signaling may be linked to a more aggressive phenotype with increased reliance on CHK1 signaling in pancreatic NEC. In our cohort, activating mutations in *APC* or *CTNNB1* were identified in 3 of 12 pancreatic NEC cases, two of which belonged to the *CHEK1*-low group, complicating straightforward interpretation of canonical WNT signaling activity based on *AXIN2* expression (Supplementary Table S7). Therefore, it is difficult at present to assess the extent to which *LRP8* expression contributes to WNT pathway activation in clinical specimens.

As observed for *CHEK1*, *LRP8* expression showed strong concordance between TPM and qPCR measurements (r = 0.95; *P* < 0.0001; Supplementary Fig. S7E). We next evaluated *LRP8* expression across multiple tumor regions within a single case (NE004), which was defined as a *CHEK1*- and *LRP8*-high case in this study. All regions exhibited elevated *LRP8* expression compared with six *LRP8*-low cases (Supplementary Fig. S7F). As with *CHEK1*, *LRP8* exhibited greater intergroup than intragroup variability (*P* = 0.001; η^2^ = 0.80; ICC = 0.72; Fig. 8D), indicating that its expression heterogeneity could partly reflect differences among phylogenetic groups. However, closer examination at the phylogenetic group level revealed a distinct expression pattern of *LRP8* compared with *CHEK1*, with some groups (e.g., group C, characterized by low *CHEK1* expression) showing high *LRP8* expression whereas others (e.g., group H, with high *CHEK1* expression) did not (Figs. 4D and 8D). Notably, both lesions in group C corresponded to metastatic sites, suggesting that this distinct expression pattern may be influenced by the metastatic context. Consistent with this observation, *LRP8* expression was significantly higher in metastatic lesions than in primary tumors (Fig. 8E). This pattern was not observed for *CHEK1*, *CDC6*, or *CLSPN* (Fig. 4A; Supplementary Fig. S3A). Despite this divergence, 5 of the 10 regions with the highest *CHEK1* expression overlapped with those with the highest *LRP8* expression, indicating partial spatial co-localization (Supplementary Fig. S7F). These co-high expression regions showed significantly higher *MKI67* expression than regions with low expression of both genes or high expression of either gene alone (Fig. 8F).

## Discussion

In this study, we identify *CHEK1* as a promising therapeutic target in GIS-NEC. Pharmacologic inhibition of *CHK1* at low nanomolar concentrations (≤50 nmol/L) impaired viability across multiple GIS-NEC cell lines. These results indicate a functional dependency on CHK1-mediated checkpoint signaling in a subset of GIS-NEC models, in line with clinical activity reported for CHK1 inhibitors in *TP53*-mutant malignancies such as HGSOC (21, 22). However, CHK1 inhibitors show limited efficacy in SCLC (20), despite similar genomic features. Such discrepancies suggest that *TP53* status alone is insufficient to determine checkpoint dependency. Prior studies have shown that CHK1 dependency varies across tissue contexts (53). Our findings similarly demonstrate organ-specific heterogeneity in *CHEK1*-associated biology within GIS-NEC. Although *TP53* mutations are frequently observed in GIS-NEC, sensitivity to CHK1 inhibition varied across cell lines. Moreover, *CHEK1* expression was not prognostic in the overall GIS-NEC cohort or in gastric NEC, whereas it was significantly associated with poor outcomes in pancreatic NEC. In pancreatic NEC, *CHEK1* expression was strongly correlated with canonical replication stress regulators, *CDC6* and *CLSPN*. These genes were coordinately upregulated in *CHEK1*-high tumors, suggesting activation of a replication stress program. In contrast, such coordinated gene expression was not observed in gastric NEC. These findings indicate that CHK1 dependency is likely shaped by organ-specific biological contexts, potentially reflecting differences in replication stress programs, rather than *TP53* mutation status alone.

Our data further suggest that specific signaling pathways influence CHK1 dependency. Screening analyses identified WNT signaling as a candidate pathway associated with resistance to CHK1 inhibition. Notably, pancreatic NEC cells A99 exhibited limited sensitivity to CHK1 inhibition despite evidence of replication stress activation. This result indicates that CHK1 dependency can be modulated by compensatory signaling pathways, such as WNT–β-catenin–driven MYC activation. Although direct regulatory interactions between CHK1 and WNT signaling remain incompletely defined, accumulating evidence supports functional interplay between WNT signaling and replication stress pathways. WNT–β-catenin signaling activates MYC-dependent transcriptional programs (54), and MYC has been implicated in promoting replication origin firing and replication stress (55, 56). These observations suggest that WNT–MYC signaling may increase reliance on CHK1-mediated checkpoint pathways and modulate CHK1 dependency in a context-dependent manner. WNT inhibition suppressed *MYC* expression and reduced *E2F* target gene expression involved in DNA replication and checkpoint control, with *CDC6* being most strongly affected, suggesting impaired origin licensing. Combined inhibition of WNT and CHK1 further exacerbated replication stress, as evidenced by increased γH2AX and pRPA32, and resulted in synergistic antitumor effects. Collectively, these results indicate that WNT signaling contributes to resistance to CHK1 inhibition and that blockade of WNT signaling can unmask CHK1 dependency. These findings provide a rationale for combined targeting of WNT and CHK1 in GIS-NEC.

These concepts are summarized in the model shown in Fig. 8G. At baseline (Fig. 8G-a), high WNT activity sustains *MYC*-driven transcriptional programs, supporting replication progression. WNT inhibition (Fig. 8G-b) reduces E2F output and replication gene expression, impairing origin licensing. CHK1 inhibition under these conditions (Fig. 8G-c) induces replication fork collapse and cell death, which is further amplified by concurrent WNT blockade.

We also identified *LRP8*, a WNT-related receptor, as preferentially upregulated in *CHEK1*-high pancreatic NEC. Prior studies have implicated *LRP8* in WNT-related signaling, as well as in cell migration and adhesion (52, 57), supporting a role in tumor progression. In line with these reports, *LRP8* expression was significantly higher in metastatic lesions than in primary tumors. In addition, primary tumors showed a broad range of *LRP8* expression, with some regions exhibiting levels comparable with those observed in metastatic lesions. These regions partially overlapped with *CHEK1*-high regions. All five overlapping regions among the top 10 regions for each were derived from primary tumors (Supplementary Fig. S7G). This partial overlap suggests a potential interaction between *LRP8*-associated programs and *CHEK1*-mediated replication stress signaling. These overlapping regions exhibited significantly higher *MKI67* expression compared with other regions. Furthermore, cases with concurrent high expression of *CHEK1* and *LRP8* showed significantly poorer prognosis compared with other cases, including those with low or single-high expression. These findings suggest that LRP8 may contribute to tumor progression not only by promoting metastasis but also potentially through interactions with replication stress signaling pathways.

This study has several limitations. The relatively small cohort size precluded direct comparison between cases with concurrent high expression of *CHEK1* and *LRP8* and those with high expression of either gene alone, limiting the ability to assess whether the observed prognostic effect is specific to concurrent high expression. In addition, the functional role of LRP8 and its mechanistic relationship with WNT signaling were not directly investigated and therefore remain to be elucidated. Moreover, the lack of *in vivo* validation demonstrating that WNT pathway inhibition can overcome prexasertib resistance is an important limitation. Although our *in vitro* findings support a functional link between WNT signaling activation and resistance to CHK1 inhibition, the proposed model remains hypothetical in the absence of *in vivo* evidence. Future studies using xenograft- or patient-derived tumor models will be required to determine whether WNT pathway blockade can restore sensitivity to prexasertib *in vivo*. Spatial expression analyses may also be influenced by regional differences in tumor cellularity. However, *CHEK1*, *CDC6*, and *CLSPN* were not upregulated in distant metastatic lesions with high tumor content, suggesting that tumor purity is unlikely to be the primary driver of the observed expression heterogeneity and that the findings likely reflect underlying biological variation. Because phylogenetic group assignment was available only for the multiregion pancreatic NEC case analyzed in our previous study, comparable phylogeny-based analyses could not be performed in the remaining cases. Validation in additional multiregion pancreatic NEC cases will therefore be required to determine the generalizability of these findings. Finally, the age distribution of our pancreatic NEC cohort was comparable with that reported in a large SEER-based study (40), and the cohort included both Asian and White patients. Nevertheless, the limited sample size warrants validation of our findings in larger, independent cohorts with broader demographic and clinical diversity.

Our findings define *CHEK1* as a context-dependent therapeutic target in GIS-NEC and establish the WNT–MYC–CHK1 axis as a mechanistically linked and therapeutically actionable vulnerability, particularly in pancreatic NEC. These results provide a rationale for combination strategies targeting both CHK1 and WNT–MYC signaling pathways and underscore the importance of lineage-specific regulatory networks in shaping therapeutic response.

## Supplementary Material

Supplementary Methods Supplementary Methods and References

Supplementary Figure S1 Supplementary Figure S1. CHEK1 is highly expressed in NEC compared to NET.

Supplementary Figure S2 Supplementary Figure S2. CHEK1 expression is associated with poor prognosis in pancreatic NEC.

Supplementary Figure S3 Supplementary Figure S3. Spatial co-localization of CHEK1, CDC6, and CLSPN expression within pancreatic NEC tumors.

Supplementary Figure S4 Supplementary Figure S4. Expression of key transcription factors in GIS-NEC cell lines.

Supplementary Figure S5 Supplementary Figure S5. CHK1 inhibition suppresses cell cycle progression in GIS-NEC cell lines.

Supplementary Figure S6 Supplementary Figure S6. Expression of DNA replication–related genes and cell cycle associated genes in GIS-NEC cell lines.

Supplementary Figure S7 Supplementary Figure S7. LRP8 expression in pancreatic NEC and association with CHEK1 and clinical aggressiveness.

Supplementary Table S1 Supplementary Table S1. Cell lines used in this study.

Supplementary Table S2 Supplementary Table S2. Reagents and antibodies used in this study.

Supplementary Table S3 Supplementary Table S3. TP53 mutation status of GIS-NEC cell lines.

Supplementary Table S4 Supplementary Table S4. CHEK1 expression in cases with GIS-NEN.

Supplementary Table S5 Supplementary Table S5. Sequences of primers used in this study.

Supplementary Table S6 Supplementary Table S6. Clinicopathological characteristics of cases with GIS-NEC stratified by CHEK1 expression.

Supplementary Table S7 Supplementary Table S7

Supplementary Table S8 Supplementary Table S8

## Data Availability

All raw data for this study are available from the corresponding author upon request.
