# Supplementary material for: Dual CHK1 and WNT Inhibition as a Synergistic Therapy for Gastrointestinal Neuroendocrine Carcinoma
Source: Cancer Res Commun. 2026 Oct 19;6(10):2434–50. doi: 10.1158/2767-9764.CRC-26-0351 (PMC13678627; doi:10.1158/2767-9764.CRC-26-0351)
Supplement: Supplementary file 1 [file crc-26-0351_supplementary_figure_s1_suppsf1.pdf]

Supplementary Figure S1

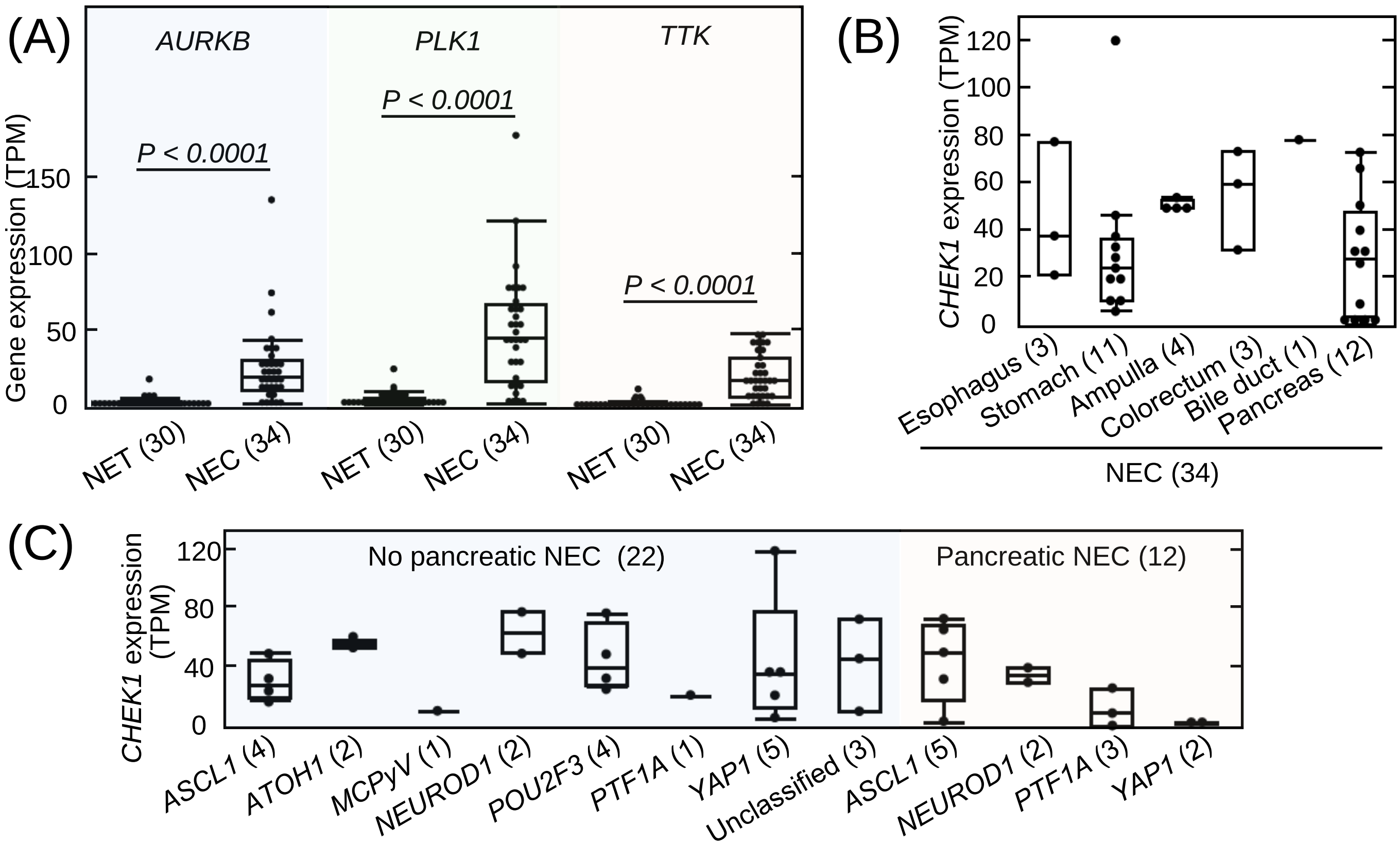

Supplementary Figure S1. *CHEK1* is highly expressed in NEC compared to NET.
A, Expression levels of *AURKB* (blue background), *PLK1* (green background), and *TTK* (red background) mRNA in tumor tissues from 64 cases with GIS-NEN were measured by RNA sequencing.
B, Expression levels of *CHEK1* mRNA according to primary tumor site were assessed by RNA sequencing in 34 cases with NEC.
C, Expression levels of *CHEK1* mRNA were compared across molecular subtypes in cases of non-pancreatic NEC (blue background) and pancreatic NEC (red background). Data are presented as box-and-whisker plots, in which the central line indicates the median, the box represents the interquartile range (25th to 75th percentile), and the whiskers extend from the 5th to the 95th percentile. P values were calculated using the Mann-Whitney U test (**A**).
