# Supplementary material for: Dual CHK1 and WNT Inhibition as a Synergistic Therapy for Gastrointestinal Neuroendocrine Carcinoma
Source: Cancer Res Commun. 2026 Oct 19;6(10):2434–50. doi: 10.1158/2767-9764.CRC-26-0351 (PMC13678627; doi:10.1158/2767-9764.CRC-26-0351)
Supplement: Supplementary file 2 [file crc-26-0351_supplementary_figure_s2_suppsf2.pdf]

Supplementary Figure S2

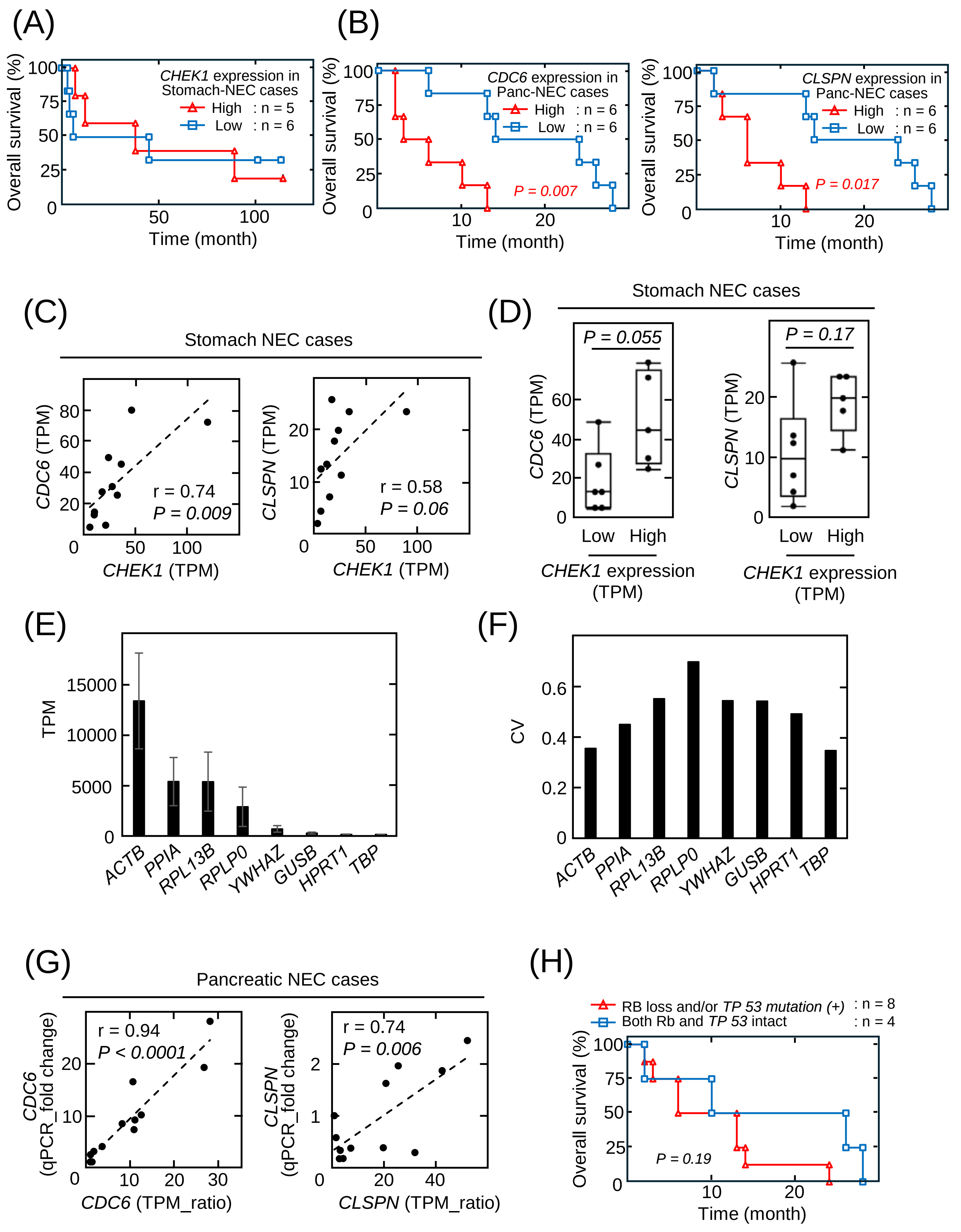

Supplementary Figure S2. High *CHEK1* expression is associated with poor prognosis in pancreatic NEC.

A, The relationship between *CHEK1* expression and overall survival in cases with stomach NEC.
B and **C**, The relationship between *CDC6* (**B**) and *CLSPN* (**C**) expression and overall survival in cases with pancreatic NEC.
C, Scatter plots showing the correlation between *CHEK1* (X-axis) and *CDC6* (Y-axis) in the left panel, and between *CHEK1* (X-axis) and *CLSPN* (Y-axis) in the right panel, in stomach NEC cases. The dotted black line represents the linear fit.
D, Comparison of *CDC6* (left) and *CLSPN* (right) mRNA expression between *CHEK1*-low and *CHEK1*-high groups in stomach NEC cases. Results are presented as box-and-whisker plots, where the median is indicated by a line, the box represents the 25th to 75th percentiles, and the whiskers show the 5th to 95th percentiles. P values were calculated using the Mann-Whitney U test.
E and F, TPM values (**E**) and coefficients of variation (CVs) (**F**) of candidate reference genes in 12 pancreatic NEC cases based on RNA-seq data.
G, Scatter plots showing the correlation between *CDC6* expression (left panel) and *CLSPN* expression (right panel) measured by RNA sequencing (X-axis) and by qPCR (Y-axis). RNA-seq expression is shown as the ratio relative to the lowest-expressing case, and qPCR expression is presented as fold change normalized to *ACTB*. The dotted black line indicates the linear fit.
H, The relationship between overall survival and Rb expression and *TP53* mutation status in pancreatic NEC cases. The log-rank test was used for statistical analysis (**A**, **B**, and **H**). Pearson's correlation coefficient (r) and the corresponding P value are indicated on each plot (**C** and **G**).
