# Supplementary material for: Dual CHK1 and WNT Inhibition as a Synergistic Therapy for Gastrointestinal Neuroendocrine Carcinoma
Source: Cancer Res Commun. 2026 Oct 19;6(10):2434–50. doi: 10.1158/2767-9764.CRC-26-0351 (PMC13678627; doi:10.1158/2767-9764.CRC-26-0351)
Supplement: Supplementary file 3 [file crc-26-0351_supplementary_figure_s3_suppsf3.pdf]

Supplementary Figure S3

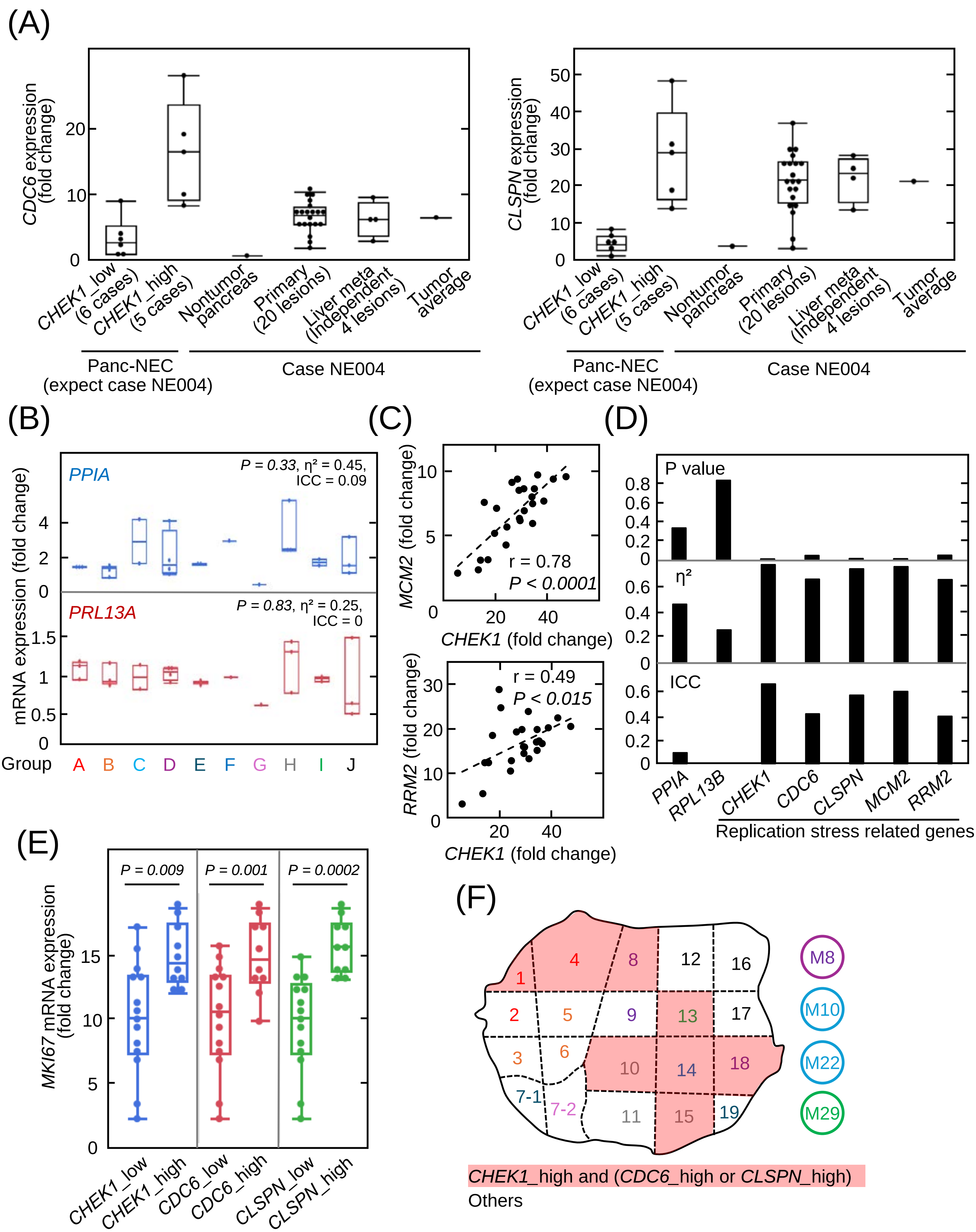

Supplementary Figure S3. Spatial co-localization of *CHEK1*, *CDC6*, and *CLSPN* expression within pancreatic NEC tumors.

A, *CDC6* expression (left panel) and *CLSPN* expression (right panel) were measured by qPCR and normalized to *ACTB*. Fold change is shown for 11 GIS-NEC cases and 24 tumor regions from Case NE004.

B, Expression levels of *PPIA* (top panel) and *PRL13A* (bottom panel) were measured by qPCR, normalized to *ACTB*, and presented as fold change relative to adjusted normal pancreas. Tumor regions were grouped into A–J shown in **Fig. 3C**.

C, Scatter plots showing the correlation between *CHEK1* (X-axis) and *MCM2* (Y-axis) in the top panel, and *CHEK1* (X-axis) and *RRM2* (Y-axis) in the bottom panel, in 24 tumor regions from Case NE004. The dotted black line represents the linear fit. Pearson's correlation coefficient (r) and the corresponding P value are indicated.

D, Summary of P values (top panel), effect sizes (η^2; middle panel), and intraclass correlation coefficients (ICCs; bottom panel) for all analyzed genes across phylogenetically defined groups.

E, *MKI67* expression were measured by qPCR, normalized to ACTB, and compared across groups stratified by *CHEK1*, *CDC6*, and *CLSPN* expression levels.

F, Among the 24 tumor regions of Case NE004, the eight regions that were among the top 10 for *CHEK1* expression and also among the top 10 for *CDC6* or *CLSPN* expression are highlighted in red.
