# Supplementary material for: Dual CHK1 and WNT Inhibition as a Synergistic Therapy for Gastrointestinal Neuroendocrine Carcinoma
Source: Cancer Res Commun. 2026 Oct 19;6(10):2434–50. doi: 10.1158/2767-9764.CRC-26-0351 (PMC13678627; doi:10.1158/2767-9764.CRC-26-0351)
Supplement: Supplementary file 4 [file crc-26-0351_supplementary_figure_s4_suppsf4.pdf]

Supplementary Figure S4

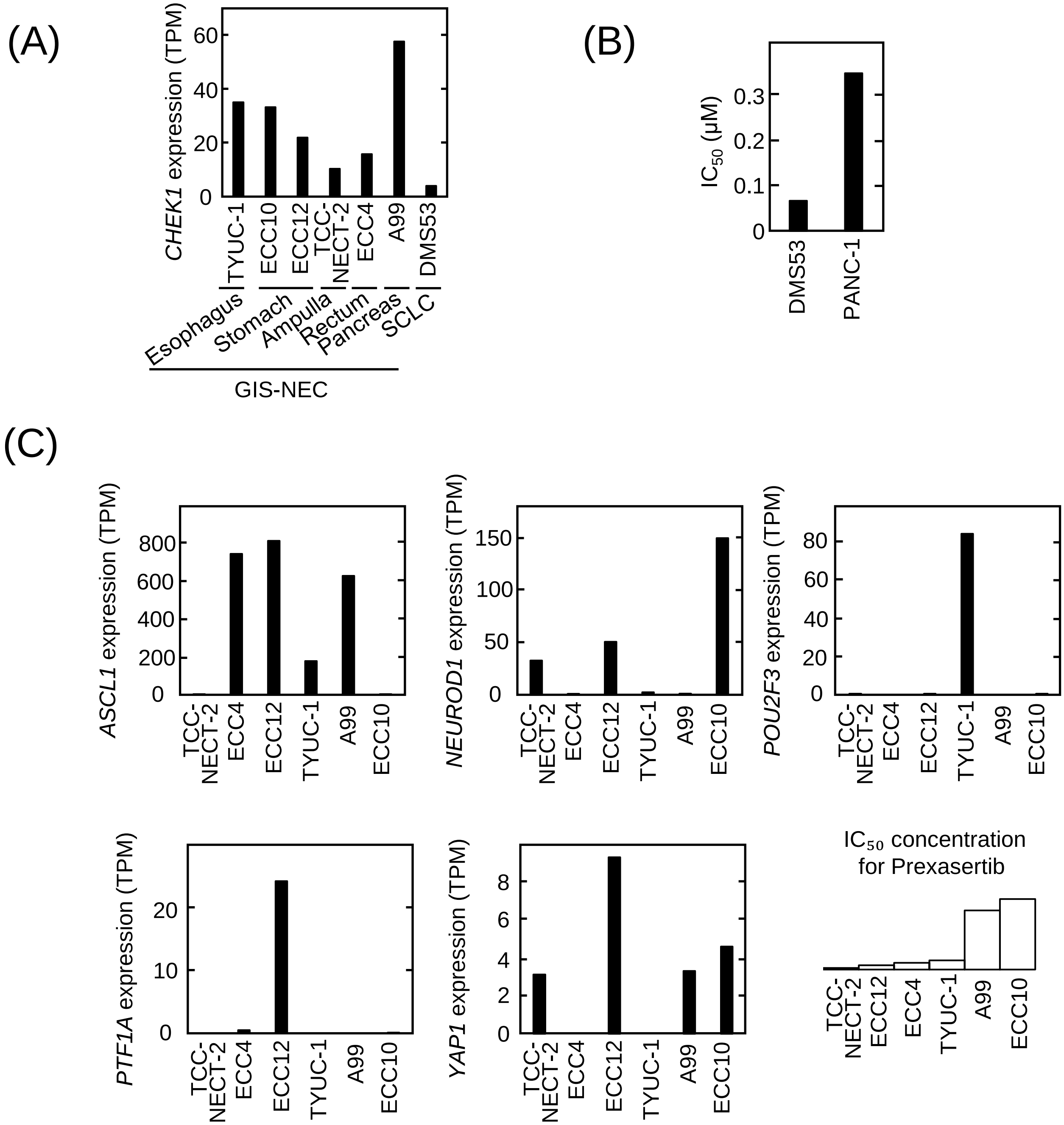

Supplementary Figure S4. Expression of key transcription factors in GIS-NEC cell lines.
A, Expression levels of *CHEK1* mRNA were measured by RNA sequencing in six GIS-NEC cell lines and one small cell lung cancer (SCLC) cell line.
B, IC_{50} values for prexasertib in the indicated cell lines.
C, Expression levels of *ASCL1*, *NEUROD1*, *POU2F3*, *PTF1A*, and *YAP1* mRNA in six GIS-NEC cell lines were quantified by RNA sequencing.
