# Supplementary material for: Dual CHK1 and WNT Inhibition as a Synergistic Therapy for Gastrointestinal Neuroendocrine Carcinoma
Source: Cancer Res Commun. 2026 Oct 19;6(10):2434–50. doi: 10.1158/2767-9764.CRC-26-0351 (PMC13678627; doi:10.1158/2767-9764.CRC-26-0351)
Supplement: Supplementary file 5 [file crc-26-0351_supplementary_figure_s5_suppsf5.pdf]

Supplementary Figure S5

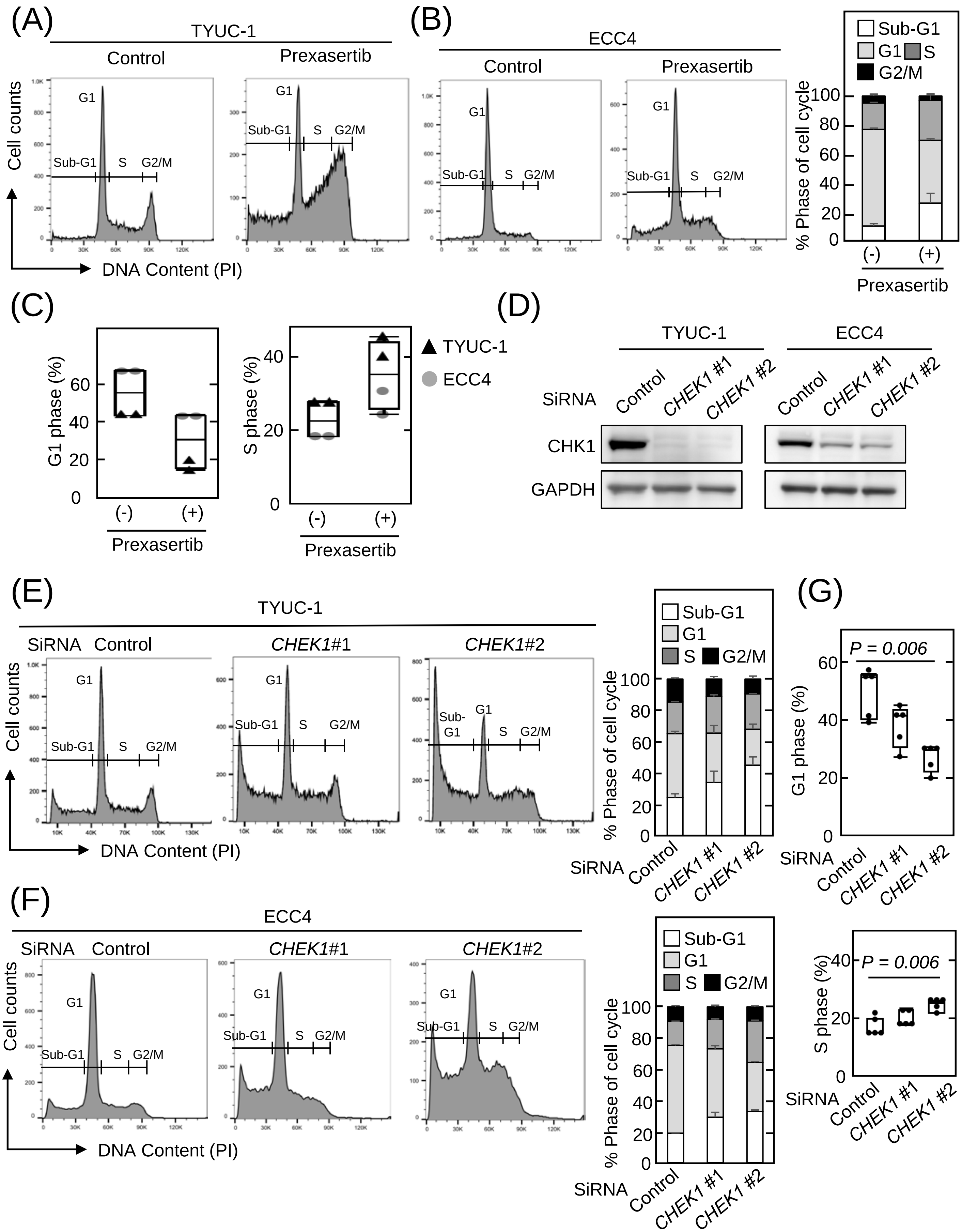

Supplementary Figure S5. CHK1 inhibition suppresses cell cycle progression in GIS-NEC cell lines.

A and **B**, Cell cycle analysis of TYUC-1 (A) and ECC4 (B) cells treated with prexasertib at the IC_{50} concentration was performed by flow cytometry 24 hours after treatment.
C, Cell cycle analysis data from independent experiments using TYUC-1 and ECC4 cell lines shown in Fig. 5A and Supplementary Fig. S5A and S5B were combined. The percentages of cells in the G1 phase (left panel) and S phase (right panel) are shown.
D, TYUC-1 cells and ECC4 cells were transfected with 25 nM control or *CHEK1* siRNA. Protein lysates were collected at 72 hours (TYUC-1) and 120 hours (ECC4) post-transfection and probed by immunoblotting using anti-CHK1 and anti-GAPDH antibodies. GAPDH served as a loading control.
E and **F**, Cell cycle analysis was performed in TYUC-1 cells (**E**) and ECC4 cells (**F**) transfected with 25 nM control or CHEK1 siRNA. Analyses were conducted 72 hours post-transfection. The percentages of cells in each cell cycle phase are presented as means ± SD from two independent experiments (right panels).
G, Cell cycle analysis data from independent experiments using TYCU-1 and ECC4 cells shown in Supplementary Fig. S5E and F were combined. The percentages of cells in the G1 phase (top panel) and S phase (bottom panel) were plotted. Data are presented as box-and-whisker plots, where the horizontal line indicates the median, the box represents the interquartile range (25th to 75th percentiles), and the whiskers denote the 5th to 95th percentiles (**C** and **G**). P values were calculated using the Kruskal-Wallis test (**G**) and the Mann-Whitney U test (**C**).
