# Supplementary material for: Dual CHK1 and WNT Inhibition as a Synergistic Therapy for Gastrointestinal Neuroendocrine Carcinoma
Source: Cancer Res Commun. 2026 Oct 19;6(10):2434–50. doi: 10.1158/2767-9764.CRC-26-0351 (PMC13678627; doi:10.1158/2767-9764.CRC-26-0351)
Supplement: Supplementary file 6 [file crc-26-0351_supplementary_figure_s6_suppsf6.pdf]

Supplementary Figure S6

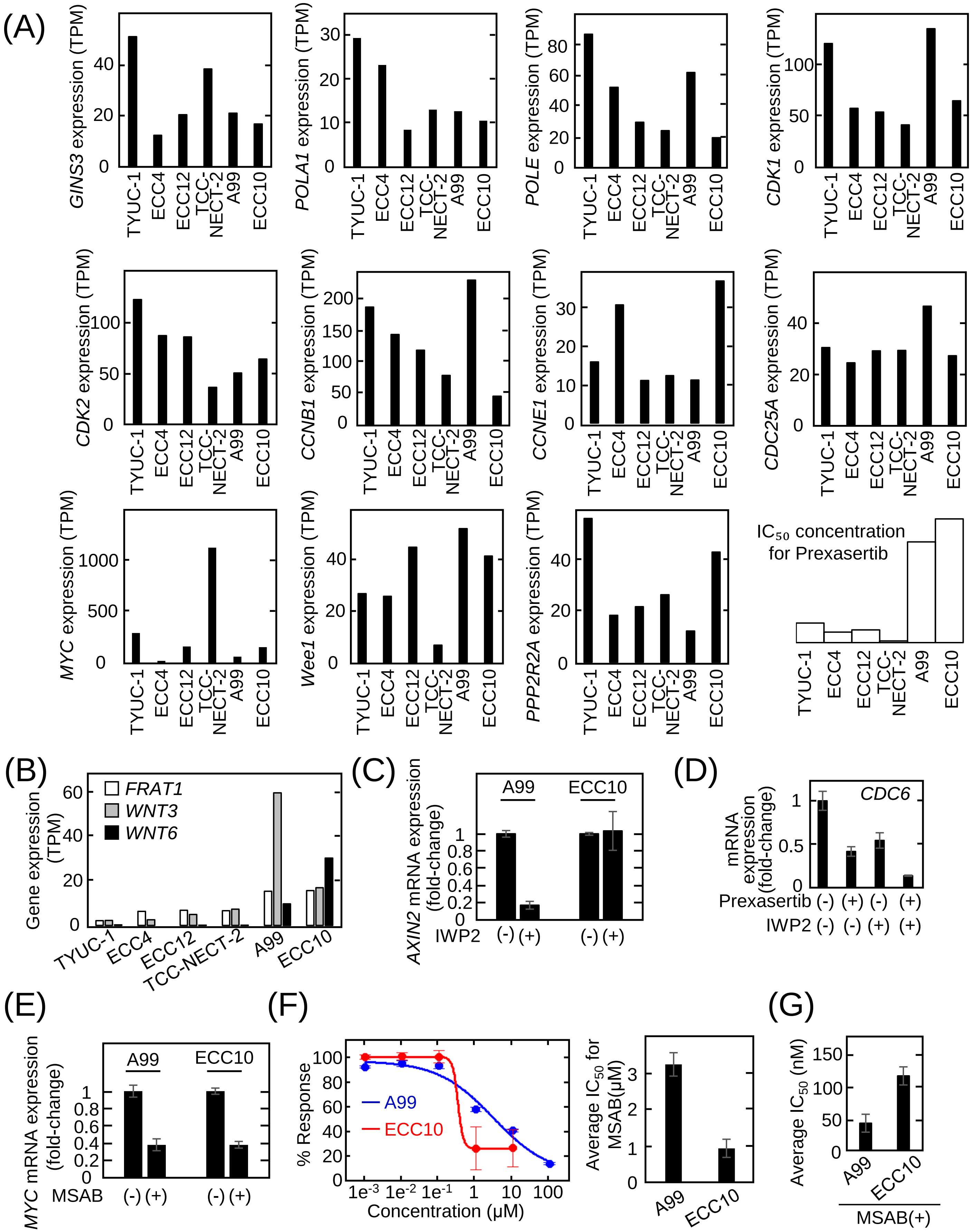

Supplementary Figure S6. Expression of DNA replication–related genes and cell cycle–associated genes in GIS-NEC cell lines.

A, mRNA expression levels of *GINS3*, *POLA1*, *POLE*, *CDK1*, *CDK2*, *CCNB1*, *CCNE1*, *CDC25A*, *MYC*, *Wee1*, and *PPP2R2A* in six GIS-NEC cell lines were quantified by RNA sequencing.

B, Expression levels of the indicated genes in 6 GIS-NEC cell lines were measured by RNA sequencing.

C, Expression levels of *AXIN2* in A99 cellsand ECC10 cells treated ± 10 μM IWP2 for 48 h were measured by quantitative RT-PCR and normalized to *GAPDH*.

D, *CDC6* mRNA levels in A99 cells treated with prexasertib (24 h), IWP2 (48 h), or both were measured by qRT-PCR and normalized to *GAPDH*. Data show fold change relative to untreated cells, mean ± SD of three independent experiments.

E, Expression levels of *MYC* in A99 cells and ECC10 cells treated ± 10 μM MSAB for 36 h were measured by quantitative RT-PCR and normalized to *GAPDH*.

F, Viability of A99 and ECC10 cells after MSAB treatment. Data represent mean ± SD of three independent experiments, each with six replicate wells (left panel). IC_{50} values are shown as mean ± SD of four independent experiments (right panel).

G, IC_{50} values of prexasertib in A99 and ECC10 cells treated with prexasertib and MSAB (1 μM for A99 cells and 0.5 μM for ECC10 cells). Data are presented as mean ± SD of five independent experiments, each with six replicate wells.
