# Supplementary material for: Dual CHK1 and WNT Inhibition as a Synergistic Therapy for Gastrointestinal Neuroendocrine Carcinoma
Source: Cancer Res Commun. 2026 Oct 19;6(10):2434–50. doi: 10.1158/2767-9764.CRC-26-0351 (PMC13678627; doi:10.1158/2767-9764.CRC-26-0351)
Supplement: Supplementary file 7 [file crc-26-0351_supplementary_methods_suppsm.docx]

**Supplementary Information**

**Dual CHK1 and WNT inhibition as a synergistic therapy for gastrointestinal neuroendocrine carcinoma**

Hirokazu Kimura^1#*^, Yoshihiko Sato^1#^, Shinichi Yachida^1,2,3*^

1. Department of Cancer Genome Informatics, Graduate School of Medicine, The University of Osaka, Osaka, Japan.
2. Integrated Frontier Research for Medical Science Division, Institute for Open and Transdisciplinary Research Initiatives (OTRI), The University of Osaka, Osaka, Japan
3. Japan Agency for Medical Research and Development -Core Research for Evolutional Science and Technology (AMED-CREST), Tokyo, Japan

^#^Contributed equally.

*Corresponding author

Hirokazu Kimura, M.D., Ph.D.

Department of Cancer Genome Informatics, Graduate School of Medicine, The University of Osaka

2-2 Yamadaoka, Suita, Osaka 565-0871, Japan

E-mail: hkimura@cgi.med.osaka-u.ac.jp

Tel: +81(6)6879-3360 Fax: +81(6)6879-3369

Shinichi Yachida, M.D., Ph.D.

Department of Cancer Genome Informatics, Graduate School of Medicine, The University of Osaka

2-2 Yamadaoka, Suita, Osaka 565-0871, Japan

E-mail: syachida@cgi.med.osaka-u.ac.jp

Tel: +81(6)6879-3360 Fax: +81(6)6879-3369

**Supplemental Methods**

**Cell viability assay**

Cells were seeded in 96-well plates (Cat# 353072, Falcon) and treated 24 hours later with prexasertib or the vehicle (control). After 96 hours, cell viability was assessed using the CellTiter 96® AQueous One Solution Assay (Cat# G358A, Promega), and absorbance was measured at 490 nm (SH-9000Lab, Corona Electric).

For the experiments shown in **Fig. 4D** and **E**, prexasertib was diluted in culture medium, resulting in varying final concentrations of DMSO and PBS (0.00001–10 μM). To control for solvent effects, vehicle control wells containing corresponding volumes of DMSO and PBS were included at each concentration, and viability values were normalized to the matched controls. Six replicate wells were analyzed per concentration (n = 6). The concentration range was optimized for each cell line, and data points at the highest concentration showing non-monotonic responses were excluded from curve fitting. Experiments were independently performed twice with GIS-NEC and GIS-NET cell lines using separate plates. For PANC-1 cells, a single independent experiment was conducted, as the observed responses were consistent with previous reports (1,2). For the combination experiments shown in **Fig. 7E** and Supplementary Fig. S6G, cells were treated with prexasertib in the presence of IWP2 (10 μM) and MSAB (1 μM for A99 cells and 0.5 μM for ECC10 cells), respectively. In these experiments, prexasertib dilutions were prepared such that the final concentrations of DMSO and PBS were identical across all wells. The prexasertib and IWP2, as well as prexasertib and MSAB, combination experiments were independently repeated five times.

For the Bliss assay, four replicate wells were analyzed for each treatment condition (n = 4), and the mean cell viability was calculated for each condition. Cell viability was normalized to that of the corresponding non-treated control to obtain relative cell viability. The experiments were independently performed five times using separate plates. For each drug combination, the expected cell viability under the assumption of Bliss independence was calculated from the relative viabilities of the corresponding single-agent treatments. Bliss deviation was calculated as the difference between the observed and predicted cell viability (observed − predicted), with negative values indicating greater-than-expected inhibition.

The half-maximal inhibitory concentration (IC₅₀) values were calculated using R based on a four-parameter logistic (4PL) model. For the A99 cell line, IC₅₀ values under control conditions were comparable between **Fig. 4** and **Fig. 6**, indicating consistent drug sensitivity across experiments.

**Immunoblotting**

Experimental procedures were as described previously (3,4). Protein lysate was prepared using RIPA buffer (Cat# 188-02453, Wako, Osaka, Japan) with protease inhibitor (Cat# 04080-24, Nacalai Tesque, Kyoto, Japan) and phosphatase inhibitor (Cat# 07575-51, Nacalai Tesque, Kyoto, Japan). Antibodies used are listed in Supplementary Table S2. SuperSignal™ West Dura Extended Duration Substrate (Cat# 34075, Thermo Fisher Scientific) was employed with a ChemiDoc™ Touch Imaging System ChemiDoc imager (RRID: SCR_021693, Bio-Rad) to detect the protein signals.

**Cell cycle assay**

Harvested cells were washed with PBS and fixed in 70% ethanol 24 hours after prexasertib administration, or at 96 hours (TYUC-1 cells) and 120 hours (ECC4 cells) following siRNA transfection. After treatment with RNase A (Cat# 70856-3, Merck, Darmstadt, Germany), the cells were stained with propidium iodide (Cat# P4864, Sigma-Aldrich, St. Louis, MO) and analyzed by flow cytometry using BD FACSCanto II (RRID: SCR_018056, BD Biosciences, San Jose, CA). Cell cycle phases were determined using FlowJo software (RRID: SCR_008520, Tree Star, Ashland, OR).

**Immunofluorescence**

Tumor sections were blocked with 0.5% Blocking Reagent (Cat# FP1012, PerkinElmer, Shelton, CT) containing 0.3% Triton X-100 (MP Biomedicals, Irvine, CA) for 1 hour, followed by overnight incubation with primary antibodies and then 1-hour incubation with secondary antibodies. Nuclei were counterstained with Hoechst 33342 (1:1000) for 15 minutes. Antibody details are given in Supplementary Table S2. Imaging and analysis were performed using the same microscope and software as in the immunohistochemistry experiments. γH2AX- or cleaved caspase-3–positive cells were quantified in eight tumor areas per group.

**Small interfering RNA**

Cells were transfected with small interfering RNA (siRNA) targeting human CHEK1 (s503, s504, Cat# 4390824, Thermo Fisher Scientific) or a negative control (Silencer Select Negative Control #1, Cat# 4390843, Thermo Fisher Scientific) using Lipofectamine RNAiMAX (Cat# 13778075, Thermo Fisher Scientific) according to the manufacturer's instructions. The following target sequences were used. CHEK1 #1, 5’-CTGCATGGTATTGGAATAACT-3’; and CHEK1 #2, 5’-TGGCAACAGTATTTCGGTATA-3’.

**Others**

Quantification and analysis of band intensities of Western blots were performed by ImageJ (RRID:SCR_003070) software. In Western blotting, HSP90 or GAPDH was used as a loading control.

**References**

1.	Sen T, Tong P, Stewart CA, Cristea S, Valliani A, Shames DS, et al. CHK1 inhibition in small-cell lung cancer produces single-agent activity in biomarker-defined disease subsets and combination activity with cisplatin or olaparib. Cancer Res. American Association for Cancer Research Inc.; 2017;77:3870–84.

2.	Klomp JE, Lee YS, Goodwin CM, Papke B, Klomp JA, Waters AM, et al. CHK1 protects oncogenic KRAS-expressing cells from DNA damage and is a target for pancreatic cancer treatment. Cell Rep. Elsevier B.V.; 2021;37.

3.	Horie M, Tanaka H, Suzuki M, Sato Y, Takata S, Takai E, et al. An integrative epigenomic approach identifies ELF3 as an oncogenic regulator in ASCL1-positive neuroendocrine carcinoma. Cancer Sci. John Wiley and Sons Inc; 2023;114:2596–608.

4.	Yachida S, Wood LD, Suzuki M, Takai E, Totoki Y, Kato M, et al. Genomic Sequencing Identifies ELF3 as a Driver of Ampullary Carcinoma. Cancer Cell. Cell Press; 2016;29:229–40.
